# Synthesis, antibacterial action, and ribosome inhibition of deoxyspectinomycins

**DOI:** 10.1038/s41429-021-00408-3

**Published:** 2021-01-27

**Authors:** Suresh Dharuman, Laura A. Wilt, Jiuyu Liu, Stephanie M. Reeve, Carl W. Thompson, John M. Elmore, Dimitri Shcherbakov, Robin B. Lee, Erik C. Böttger, Richard E. Lee

**Affiliations:** 1 Department of Chemical Biology and Therapeutics, St. Jude Children’s Research Hospital, Memphis, TN USA; 2 Institut für Medizinische Mikrobiologie, Universität Zürich, Zürich, Switzerland

**Keywords:** Antibiotics, Structure-based drug design

## Abstract

Spectinomycin, an aminocyclitol antibiotic, is subject to inactivation by aminoglycoside modifying enzymes (AMEs) through adenylylation or phosphorylation of the 6-hydroxy group position. In this study, the effects of deoxygenation of the 2- and 6-hydroxy group positions on the spectinomycin actinamine ring are probed to evaluate their relationship to ribosomal binding and the antimicrobial activities of spectinomycin, semisynthetic aminomethyl spectinomycins (amSPCs), and spectinamides. To generate these analogs, an improved synthesis of 6-deoxyspectinomycin was developed using the Barton deoxygenation reaction. 6-Dehydrospectinamide was also synthesized from spectinamide **4** to evaluate the H-bond acceptor character on the C-6 position. All the synthesized analogs were tested for antibacterial activity against a panel of Gram (+) and Gram (−) pathogens, plus *Mycobacterium tuberculosis*. The molecular contribution of the 2- and 6-hydroxy group and the aryl functionalities of all analogs were examined by measuring inhibition of ribosomal translation and molecular dynamics experiments with MM/GBSA analysis. The results of this work indicate that the 6-hydroxy group, which is the primary target of AMEs, is a required motif for antimicrobial activity in current analogs. Removal of the 6-hydroxy group could be partially rescued by offsetting ribosomal binding contributions made by the aryl side chains found in the spectinamide and amSPCs. This study builds on the knowledge of the structure–activity relationships of spectinomycin analogs and is being used to aid the design of next-generation spectinomycins.

## Introduction

**Fig. 1:**
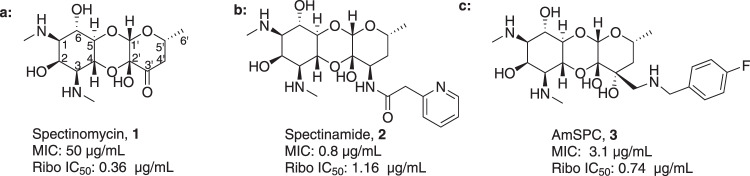
**a** Structure of spectinomycin (**1**) with activity against *M. tuberculosis* and ribosomal IC_50_ against *M. smegmatis* ribosomes. **b** Structure of spectinamide **2** with activity against *M. tuberculosis* and ribosomal IC_50_ against *M. smegmatis* ribosomes. **c** Structure of amSPC **3** with activity against *S. pneumoniae* and ribosomal IC_50_ against *M. smegmatis* ribosomes

Spectinomycin, an aminoinositol antibiotic [1, 2], is an inhibitor of bacterial protein synthesis (IC_50_ = 0.4 μg ml^−1^) with moderate broad-spectrum activity against both Gram (+) and Gram (−) bacteria (Fig. 1a). Spectinomycin binds to the interface between the minor groove of helix-34 of the 16S ribosomal RNA and the RpsE protein to inhibit protein translation [3]. Clinically, spectinomycin has been widely used to treat *Neisseria gonorrheal* infections [4] and is well tolerated at a high dosage, lacking the ototoxicity commonly associated with aminoglycoside antibiotics [5]. However, spectinomycin is subject to native efflux [6, 7], target modification [8], and enzymatic inactivation of the drug [8, 9]. Therefore, to overcome innate resistance mechanisms, we developed semisynthetic analogs of spectinomycin called spectinamides [10] and aminomethyl spectinomycins (amSPCs) [11]. The spectinamides have excellent narrow spectrum antitubercular activity, and the amSPCs have broad-spectrum activity against the common respiratory tract, sexually transmitted and biothreat bacterial pathogens (Fig. 1b, c) [12–14]. These analogs have improved bacterial accumulation, with the spectinamides overcoming efflux by the Rv1258c transporter found in *Mycobacterium tuberculosis* which transports spectinomycin [10]. While these analogs overcome the resistance from efflux pumps, they are still subject to modification by aminoglycoside modifying enzymes (AMEs) in non-mycobacterial pathogens, which limits their development for broader indications, particularly for the treatment of Gram-negative infections [15, 16]. One strategy to alleviate AME resistance is to remove the sites of inactivation found on aminoglycosides [17]. Spectinomycin is susceptible to inactivation by nucleotidyltransferases ANT(9) [18] and the bifunctional enzyme ANT(3,9), as well as phosphorylation by the APH(9) enzyme, which all modify the 6-hydroxy group position of spectinomycin [15, 16, 19]. Previously, several deoxyspectinomycins were synthesized by researchers at Hoffmann-La Roche Inc. and were found to be microbiologically inactive [20–22]. However, it was unknown if the inactivity is due to lack of ribosomal binding or poor accumulation. As part of our systematic approach to develop the structure–activity relationship (SAR) around the spectinomycin ring, we chose to reinvestigate the antimicrobial properties of deoxyspectinomycin analogs and to evaluate their ability to inhibit bacterial ribosomes, which has not been previously reported.

**Fig. 2:**
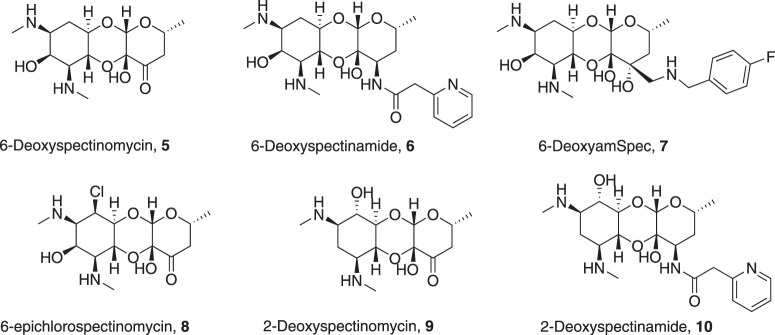
Proposed analogs of deoxyspectinomycins to overcome aminoglycoside modifying enzyme resistance

**Fig. 3:**
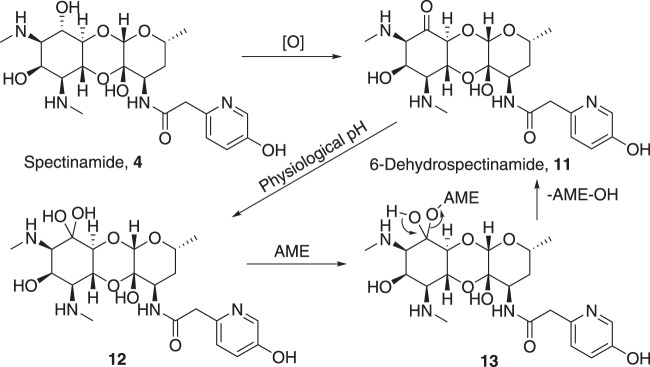
Proposed mechanism for 6-dehydrospectinamide **11** to sustain resistance from AMEs

In this study, we examine whether modifications to the 2- and 6-hydroxy groups in spectinomycin, spectinamides, and amSPCs (Fig. 2) can be tolerated, how modification or removal of these groups affects antibacterial potency, ribosome inhibition, and cellular accumulation, and finally, computationally examine the relative binding contributions of these motifs in the ribosome binding site. We report an improved synthesis of 6-deoxyspectinomycin using Barton’s radical deoxygenation reaction as the key step [23] and demonstrate an efficient synthesis of 6- and 2-deoxyspectinamides and 6-deoxyaminomethyl spectinomycin (6-deoxy-amSPC) analogs [21]. 6-*epi-*chlorospectinomycins and 6-dehydrospectinamide were also synthesized to examine hydrogen bonding acceptor character to the ribosome (Fig. 3). The antimicrobial activity and cellular accumulation of the analogs were determined, and the energetic contribution of key binding interactions was examined computationally. Results from these studies are combined to provide a holistic picture of the value of the 2- and 6-hydroxy groups to spectinomycin binding and antimicrobial activity.

## Material and methods

### Chemicals, reagents, and instrumental

Spectinomycin dihydrochloride pentahydrate was purchased from Waterstone Technology (catalog number 81249, CAS number 22189-32-8, 95% in purity). All solvents used for chromatography and liquid chromatography were purchased from Aldrich. Flash column chromatography silica cartridges were obtained from Biotage Inc. Reactions were monitored by thin-layer chromatography on pre-coated Merch 60 F254 silica gel plates and visualized using UV light (254 nm). IR (neat) spectra were obtained using a Nicolet-iS5 FT-IR instrument. A Biotage FLASH column chromatography system was used to purify mixtures. ^1^H NMR spectra were recorded on a Varian INOVA-500 spectrometer or on a Bruker 400 MHz NMR spectrometer. Chemical shifts (*δ*) are reported in parts per million relative to the residual solvent peak or internal standard (tetramethylsilane), and coupling constants (*J*) are reported in hertz (Hz). High-resolution mass spectra were recorded on a Waters Xevo G2 QTOF LC–MS using ESI. Purity of the products was confirmed by UPLC/MS (the Waters Acquity). Spectral data for final compounds is given below, and data for intermediates are provided in Supplementary information.

### Chemical synthesis

#### 6-Deoxyspectinomycin dihydrochloride (**5**)

To a solution of cbz protected 6-deoxyspectinomycin **16** [20] (0.080 g, 0.136 mmol) in 1 M HCl in MeOH (3 ml) was added Pd/C (30 mg) at room temperature and the reaction mixture was hydrogenated for 30 min under H_2_ atmosphere (H_2_ Balloon). Excess catalyst was filtered off and the evaporation, washing with acetone (5 ml) and EtOAc (5 ml) gave pure product **5** as a white solid. Yield: 0.060 g, 81%; ^1^H NMR (500 MHz, D_2_O) *δ* 4.76 (s, 1H), 4.57 (s, 1H), 4.18 (t, *J* = 10.5 Hz, 1H), 4.07 (td, *J* = 11.3, 4.0 Hz, 1H), 3.92 (q, *J* = 6.6 Hz, 1H), 3.40–3.32 (m, 2H), 3.28 (s, 3H), 2.77–2.74 (m, 1H), 2.71 (s, 3H), 1.89–1.72 (m, 3H), 1.17 (d, *J* = 6.2 Hz, 3H); ^13^C NMR (125 MHz, D_2_O) *δ* 93.8, 93.4, 92.0, 68.4, 67.8, 65.2, 61.1, 59.0, 56.0, 41.5, 30.6, 30.0, 25.5, 19.6. HRMS (ESI) *m/z* calcd for C_14_H_25_N_2_O_6_ [M + H]^+^, 317.1713; found, 317.1712.

#### 6-Deoxy-3′-deoxy-3′-dihydro-3′(*R*)-[(pyridin-2-yl)acetylamino]spectinomycin dihydrochloride (**6**)

Compound **6** was synthesized analogously as **5** from **17**, as a white solid. Yield: 0.017 g (95%); IR_υmax_ (neat): cm^−1^ 3363, 2973, 1656,1561, 1467, 1166, 1064; ^1^H NMR (500 MHz, D_2_O) *δ* 8.66 (d, *J* = 5.8 Hz, 1H, Ar-*H*), 8.47 (t, *J* = 8.0 Hz, 1H, Ar-*H*), 7.95–7.84 (m, 2H, Ar-*H*), 4.90 (s, 1H), 4.56 (s, 1H), 4.26 (t, *J* = 10.4 Hz, 1H), 4.17–3.96 (m, 5H), 3.37 (d, *J* = 11.6 Hz, 2H), 2.73 (s, 3H), 2.71 (s, 3H), 2.15 (dd, *J* = 10.6, 6.7 Hz, 1H), 1.83 (t, *J* = 13.5 Hz, 2H), 1.69 (d, *J* = 14.6 Hz, 1H), 1.18 (d, *J* = 6.0 Hz, 3H); ^13^C NMR (125 MHz, D_2_O) *δ* 169.0, 149.2, 146.9, 141.4, 128.3, 125.8, 93.2, 90.2, 68.0, 67.8, 65.3, 61.6, 58.7, 55.9, 52.3, 39.2, 34.1, 30.6, 30.5, 25.4, 19.8; HRMS (ESI) *m/z* calcd for C_21_H_33_N_4_O_6_ [M + H]^+^, 437.2400; found, 437.2393.

#### 6-Deoxy-3′-dihydro-3′(*R*)-[(4-fluorobenzyl)aminomethyl]spectinomycin trihydrochloride (**7**)

Compound **7** (0.011 g, 70%) was synthesized analogously as **6** from **24**, as a white solid. Yield: 0.011 g (70%); [*α*]_D_^28^ + 0.072 (*c* 0.15, CH_3_OH); MP: 211.1–211.8; IR_υmax_ (neat): cm^−1^ 3358, 2974, 1603, 1513, 1460, 1163, 1081; ^1^H NMR (500 MHz, D_2_O) *δ* 7.46–7.43 (m, 2H, Ar-*H*), 7.17–7.14 (m, 2H, Ar-*H*), 4.67 (s, 1H), 4.58 (s, 1H), 4.27 (d, *J* = 13.5 Hz, 1H), 4.20 (d, *J* = 13.6 Hz, 1H), 4.18–4.02 (m, 2H), 3.68 (dq, *J* = 12.3, 6.1 Hz, 1H), 3.47–3.26 (m, 3H), 3.13 (d, *J* = 13.7 Hz, 1H), 2.73 (s, 3H), 2.71 (s, 3H), 2.14 (dt, *J* = 8.8, 4.3 Hz, 1H), 1.88–1.63 (m, 3H), 1.14 (d, *J* = 5.9 Hz, 3H); ^13^C NMR (125 MHz, D_2_O) *δ* 163.2 (d, *J* = 247.0 Hz, *C*-F), 132.3, 132.2, 125.9, 116.3, 116.1, 93.4, 92.5, 72.2, 67.5, 67.4, 65.0, 61.2, 58.8, 55.9, 50.8, 48.9, 40.1, 30.5, 30.2, 25.4, 19.8; ^19^F NMR (D_2_O, 375 MHz) *δ* −112.0; HRMS (ESI) *m/z* calcd for C_22_H_35_FN_3_O_6_ [M + H]^+^, 456.2510; found, 456.2510.

#### 6-*Epi*-chloro-6-deoxyspectinomycin dihydrochloride (**8**)

Compound **8** was synthesized from cbz-spectinomycin [24]. Yield: 0.012 g (42%); ^1^H NMR (500 MHz, D_2_O) *δ* 4.93 (s, 1H), 4.75–4.73 (m, 1H); 4.66 (t, *J* = 10.6 Hz, 1H), 4.44 (dd, *J* = 10.5, 3.5 Hz, 1H), 4.05–3.99 (m, 1H), 3.93–3.81 (m, 1H), 3.54 (dd, *J* = 11.0, 3.3 Hz, 1H), 2.89 (s, 3H), 2.84–2.83 (m, 1H), 2.81 (s, 3H), 1.95–1.80 (m, 2H), 1.26 (d, *J* = 6.3 Hz, 3H); ^13^C NMR (125 MHz, D_2_O) *δ* 93.8, 93.4, 91.7, 68.6, 66.5, 63.0, 61.6, 58.8, 56.8, 54.4, 41.5, 30.5, 30.2, 19.5. HRMS (ESI) *m/z* calcd for C_14_H_24_ClN_2_O_6_ [M + H]^+^, 351.1323; found, 351.1329.

#### 2-Deoxyspectinomycin dihydrochloride (**9**)

Compound **9** [21] was synthesized analogously as **5** from **20**, as a white solid. Yield: 0.010 g (80%) ^1^H NMR (500 MHz, D_2_O) *δ* 4.77 (s, 1H), 4.04 (t, *J* = 10.3 Hz, 1H), 3.96–3.88 (m, 2H), 3.74 (t, *J* = 10.2 Hz, 1H), 3.46 (t, *J* = 11.6 Hz, 1H), 3.22 (t, *J* = 10.2 Hz, 1H), 2.70 (s, 3H), 2.68 (s, 3H), 2.63–2.57 (m, 1H), 1.81–1.68 (m, 3H), 1.15 (d, *J* = 6.1 Hz, 3H); ^13^C NMR (125 MHz, D_2_O) *δ* 93.5, 93.4, 91.6, 69.9, 68.4, 68.2, 68.0, 57.3, 54.3, 41.5, 30.1, 29.7, 23.3, 19.6; HRMS (ESI) *m/z* calcd for C_14_H_25_N_2_O_6_ [M + H]^+^, 317.1713; found, 317.1713.

#### 2-Deoxy-3′-deoxy-3′-dihydro-3′(*R*)-[(pyridin-2-yl)acetylamino]spectinomycin dihydrochloride (**10**)

Compound **10** was synthesized analogously as **6** from **21**, as a white solid. Yield: 0.010 g (79%); IR_υmax_ (neat): cm^−1^ 3350, 2974, 1648, 1564, 1466, 1168, 1063; ^1^H NMR (500 MHz, D_2_O) *δ* 8.66 (d, *J* = 5.9 Hz, 1H), 8.48 (t, *J* = 8.0 Hz, 1H), 8.05–7.81 (m, 2H), 4.93 (s, 1H), 4.27–3.89 (m, 6H), 3.77 (q, *J* = 9.9, 9.2 Hz, 1H), 3.50 (td, *J* = 11.5, 4.1 Hz, 1H), 3.25 (ddd, *J* = 14.1, 10.2, 4.3 Hz, 1H), 2.72 (s, 3H), 2.71 (s, 3H), 2.68–2.56 (m, 1H), 1.86–1.68 (m, 3H), 1.18 (d, *J* = 5.7 Hz, 3H); ^13^C NMR (125 MHz, D_2_O) *δ* 166.3, 146.5, 146.4, 144.4, 138.7, 125.7, 123.1, 90.3, 87.3, 67.4, 65.6, 65.3, 54.6, 51.4, 49.5, 36.5, 31.5, 27.4, 20.9, 17.1, 11.1; HRMS (ESI) *m/z* calcd for C_21_H_33_N_4_O_6_ [M + H]^+^, 437.2400; found, 437.2401.

#### 6-Dehydro-3′-deoxy-3′-dihydro-3′(*R*)-[(5-hydroxypyridin-2-yl)acetylamino]spectinomycin trihydrochloride (**11**)

To a mixture of dimethylsulfoxide (5 ml), dry benzene (5 ml), dicyclohexyl carbodiimide (0.320 g, 1.560 mmol), and pyridinium trifluoroacetate (0.050 g, 0.260 mmol) was added **25** [25] (0.430 g, 0.520 mmol). The mixture was stirred at 50 °C for 4 h. The reaction mixture was then poured into 100 ml of ethyl acetate and 100 ml of water with stirring, the insoluble dicyclohexyl urea was removed by filtration, and the ethyl acetate phase was washed with water and dried over Na_2_SO_4_. After removing solvent, the residue was purified by C18 chromatography (H_2_O/CH_3_CN), and 200 mg (47%) white solid was obtained as 6-dehydro-cbz-**25**. 6-Dehydro-cbz-**25** (157 mg, 0.190 mmol) was mixed with 10% Pd/C (20 mg) and 1 M HCl in MeOH (1 ml) in MeOH (10 ml), the mixture was hydrogenated at room temperature for 4 h. Pd/C was removed and then the filtrate was dried under vacuum to yield **11** as a white solid. Yield: 0.080 g (73%); IR_υmax_ (neat): cm^−^^1^ 3254, 2923, 2850, 1651, 1552, 1495, 1168, 1067;^1^H NMR (400 MHz, D_2_O) *δ* 8.29 (d, *J* = 2.8 Hz, 1H), 8.02–7.93 (m, 1H), 7.75 (d, *J* = 8.9 Hz, 1H), 5.14–5.04 (m, 1H), 4.61–4.49 (m, 1H), 4.30–4.03 (m, 6H), 3.60 (dd, *J* = 11.2, 3.2 Hz, 1H), 3.51–3.35 (m, 1H), 2.94–2.84 (m, 6H), 2.03–1.85 (m, 1H), 1.88–1.71 (m, 1H), 1.29 (dd, *J* = 6.1, 4.2 Hz, 3H); ^13^C NMR (125 MHz, D_2_O) *δ* 169.6, 169.5, 154.9, 140.3, 133.5, 133.5, 129.2, 129.2, 129.0, 128.9, 94.1, 93.9, 93.2, 93.0, 92.2, 92.1, 90.2, 90.0, 89.8, 71.8, 70.8, 69.9, 68.4, 67.2, 64.7, 61.8, 60.1, 58.3, 57.8, 54.0, 52.2, 48.8, 38.2, 34.1, 30.8, 30.7, 30.2, 30.0, 19.8, 19.8. HRMS (ESI) *m/z* calcd for C_21_H_31_N_4_O_8_ [M + H]^+^, 467.2142; found, 467.2144.

### Molecular modeling

#### Mycobacterial RNA-RpsE protein complex generation

**Fig. 4:**
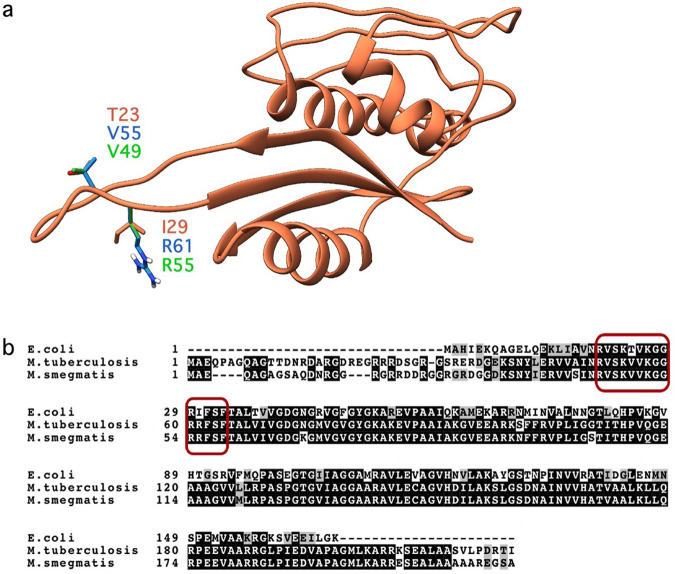
Alignment of *E. coli, M. tuberculosis*, and *M. smegmatis* RpsE protein. **a** The alignment of the protein structures of the RpsE protein from *E. coli* from PDB ID 2QOU [26] (orange), the homology model from *M. tuberculosis* (blue), and *M. smegmatis* (green) developed in Schrödinger using Prime [27, 28]. Residues that are different in the RpsE protein loop between species are shown as sticks and are labeled. **b** The sequence alignment of the RpsE proteins [49, 50]. Black highlights represent identical residues, gray highlights represent similar residues, and the red boxes highlight the RpsE protein loop

The molecular docking and molecular dynamics studies of spectinomycin and spectinomycin analogs were performed using a comparative model of the *M. tuberculosis* RNA-RpsE protein complex prepared using the *Escherichia coli* complex as a template. The Mycobacterial RpsE proteins (*M. tuberculosis* and *Mycobacterium smegmatis*) have a high degree of homology, with 89% sequence similarity. The differences in the RpsE protein sequences lie outside the spectinomycin binding site on the RpsE loop (Fig. 4). Therefore, the *M. tuberculosis* RpsE protein sequence was used to create a Mycobacterial RNA-RpsE protein complex for molecular modeling of both species. The *E. coli* RpsE protein structure (PDB ID: 4V56 (2QOU)) served as the structural template to generate the model, 55% homology [26]. The model was created using Schrödinger’s Prime program [27, 28] and was validated in PROCHECK [29] with 2.7% residues allowed and 0% disallowed (data not shown). A sequence alignment of RpsE proteins from the Mycobacteria and *E. coli* revealed two amino acid differences located in the RpsE protein loop in the spectinomycin binding site: V55T and R61I (Fig. 4). Despite variations in the RpsE protein, the rRNA sequence found in the spectinomycin binding site is highly conserved between Mycobacteria and *E. coli*. To reduce the computational cost of subsequent molecular docking and molecular simulations, the complex model was truncated to a 20 Å sphere around the spectinomycin binding site. The Mycobacterial RNA-RpsE complex was protonated at pH 7.0 using PROPKA and was energy minimized with the OPLS3 force field [30, 31].

#### Ligand preparation

SMILES strings for spectinomycin and analogs were used to render 3D conformations of the compounds in Schrödinger. Minimum energy conformations of each analog were generated by LigPrep using the OPLS3 force field [30]. Specified chiralities were retained and the ionization states at a target pH 7.0 ± 2.0 were generated using Epik [32].

#### Receptor grid generation

For the molecular docking of spectinomycin and analogs, a receptor grid was generated using Schrödinger’s Glide program [33–35]. A cubic box was positioned in the center of the spectinomycin binding site. The inner box was set to 10 × 10 × 10 Å and the outer box was 30 × 30 × 30 Å. This larger box was designed to permit the docking of spectinamides and amSPCs in the binding site. The spectinomycin binding site determined by X-ray crystallography (PDB ID 2QOU) [26] revealed a series of hydrogen bond interactions from the rRNA nucleotides. Therefore, hydrogen bonding constraints were generated for residues G1064, C1066, G1068, A1191, C1192, and G1193 for a data-driven docking approach.

#### Glide standard precision molecular docking

All conformations generated from LigPrep were docked using Schrödinger’s Glide program [33–35]. Ligand sampling was flexible, and docked conformers were required to match at least one hydrogen bonding interaction from the constraints generated in the receptor grid. To soften the potential for nonpolar parts of the ligands, van der Waals (VDW) radii were scaled using a scaling factor of 0.80 for ligand atoms with a partial charge < 0.15 (default Glide settings). Post-docking minimization was performed on all poses generated by Glide using the OPLS3 force field prior to the assignment of Glide docking scores. Ligand poses with the highest ranked Glide scores were used to evaluate binding. The highest ranked analogs, **1** (spectinomycin), **2**, **5**, **6**, **9**, and **10**, were advanced to molecular dynamics simulations.

#### Molecular dynamics

All molecular dynamic simulations were performed using AMBER18 [36] as previously described [25]. Briefly, the ff14SB and OL3 force field was used for the RNA and protein parameterization and the general Amber force field was used for parameterization of the ligand [37–39]. Restrained electrostatic potential charges within the ANTECHAMBER module in AMBER were applied to the positively charged spectinomycin, **2**, **5**, **6**, **9**, and **10** analogs. Sodium ions were applied to neutralize the charge of the system. The complex was solvated in a TIP3P octahedron box with a 10 Å boundary. An initial energy minimization of the solvated complex was performed with the complex fixed for 1000 steps, followed by a second step of energy minimization without restraints for 2500 steps. The minimized complex was equilibrated to 300 K over 250,000 steps for 500 ps with weak restraints applied to the complex (Fig. S1). The equilibrated structure was simulated for 20 ns with the NPT ensemble at 300 K with terminal residues harmonically restrained (Fig. S1). The energetic contributions during the MD simulations were calculated by the MM/GBSA [40] method using 1000 frames from the last 2 ns of the MD ensemble. Lastly, the free energy contributions to ligand binding were decomposed for analysis using a per-residue basis [40].

#### Ribosomal inhibition

Purified 70S *M. smegmatis* bacterial ribosomes were used in translation reactions. Firefly luciferase mRNA was produced in vitro using T7 RNA polymerase. Translation reactions were carried out as previously described [11]. The IC_50_ values represent the drug concentration that inhibits luciferase activity by 50%.

#### Bacterial strains and growth conditions

Strains were routinely grown with Mueller−Hinton broth at 37 °C with shaking at 225 rpm. Bacterial strains were obtained through ATCC or BEI Resources or from academic laboratories. The strains used were the following: *Staphylococcus aureus* ATCC 29213, *S. aureus* NRS70, *Enterococcus faecalis* ATCC 33186, *E. faecium* ATCC 19434, *Streptococcus pyogenes* ATCC 700294, *Streptococcus pneumoniae* R6, *Acinetobacter baumannii* ATCC 19606, *Pseudomonas aeruginosa* ATCC 15692, *Klebsiella pneumoniae* ATCC 700603, *Proteus mirabilis* ATCC 25933, *Stenotrophomonas maltophilia* ATCC 13637, *Enterobacter cloacae* ATCC 13047, *Staphylococcus epidermidis* ATCC 14990, *E. coli* K-12, and *E. coli* JW5503 (ΔtolC). The *M. tuberculosis* H37Rv strain was grown in Middlebrook 7H9 broth supplemented with 10% albumin-dextrose complex, 0.05% (v/v) Tween 80 at pH 7.4, (7H9/ADG pH 7.4) with shaking at 225 rpm at 37 °C.

### Antibacterial susceptibility testing

Minimum inhibitory concentrations are determined following CLSI broth microdilution guidelines [41]. Briefly, experimental compounds (in DMSO) and control spectinomycin (Sigma Aldrich, DMSO) are serially diluted, twofold, across a 96-well round bottom plate in Mueller–Hinton II broth or 7H9/ADG pH 7.4 for *M. tuberculosis* H37Rv. Equal volume of bacterial strains of 5 × 10^5^ CFU ml^−^^1^ was added to each well to give a final drug concentration starting at 200 μg ml^−1^. Plates were incubated for 18 h at 37 °C except *M. tuberculosis* H37Rv, which was incubated for 7 days at 37 °C. The MIC for all strains were determined as the lowest concentration to visually inhibit bacterial growth and are reported as the consensus of three independent experiments.

### Whole-cell accumulation assay

Methods for this assay have been optimized from previously published studies [42, 43]. *E. coli* (BW25113 for WT or JW5503 for ΔtolC) is grown at 37 °C to mid-log phase (OD600 of 0.6−0.7) in Mueller–Hinton broth. The bacteria are pelleted and washed twice with PBS before being resuspended in 3.5 ml of PBS per 100 ml of cells cultured. Cells are allowed to equilibrate for 10 min at 37 °C prior to drugging. 1 ml of *E. coli* is incubated with a final concentration of 50 μM of compound (in DMSO) shaking at 37 °C for 10 min. 800 μl of treated cells is layered over supercooled (−78 °C) silicone oil (9:1 AR20 and high-temperature silicone oil, Sigma Aldrich) and pelleted to remove cells from free compound. For lysis, the pellets were resuspended in 200 μl of HPLC grade water and subjected to three freeze−thaw cycles using liquid nitrogen and a 65 °C water bath. Cell debris was separated from lysate by centrifugation, and 150 μl of extract is recovered. The pellet is resuspended in the remaining 50 μl of water, and once thoroughly resuspended, 100 μl of 5% TCA was added. The cells are once again pelleted, and lysates are combined. Lysates are pelleted at high speed for 10 min and then filtered through a 0.22 μm filter before injection for LC–MS/MS.

Samples are analyzed with a tandem Waters Acquity M Class series UPLC system and Xevo G2 QTOF tandem MS/MS with Zspray. 100 nl of extract was separated using a Phenomenex Kinetex 2.6 μm XB-C18, 100 Å (300 μm × 150 mm) column with solvent A, 0.1% formic acid in water, and solvent B, 0.1% formic acid in acetonitrile. The inlet method for these samples utilized a flow rate of 8 μl min^−1^ with the following gradient: 0−4 min, 99.9% solvent A and 0.1% solvent B; 4–5 min, 10% solvent A and 90% solvent B; 5–6 min, 99.9% solvent A and 0.1% solvent B. Tandem mass spectra were acquired with a cone voltage and collision energy optimized for each compound. High-resolution spectra were calibrated by co-infusion of 2 ng ml^−1^ leucine enkephalin lockspray (Waters). Data were quantified using Waters MassLynx software where the AUC was determined by integrating the corresponding daughter peak of the parent compound. Concentrations of the unknown compounds were determined by the linear fit of the corresponding standards. Concentrations are reported as the average of three biological replicates.

## Results and discussion

### Synthesis

**Scheme 1:**
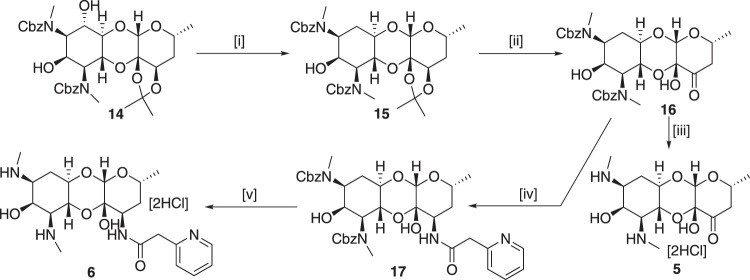
Synthesis of 6-deoxyspectinomycin **5** and 6-deoxyspectinamide **6**. Reagents and conditions: [i] (a) 1,1′-Thiocarbonyldiimidazole, DMAP, DCM, (b) Bu_3_Sn-H/AIBN, Toluene, 55% over two steps; [ii] (a) 1 M HCl in MeOH, (b) IBX, DMSO, 85% over two steps; [iii] Pd/C-H_2_, MeOH, 81%; [iv] (a) NH_4_NO_3_/CH_3_COOH, 2-picoline-borane complex, MeOH, (b) 2-Pyridylacetic acid hydrochloride, HBTU, DMF, 25% over two steps; [v] Pd/C-H_2_, MeOH, 95%

Adapting literature procedures, compound **14** [22, 44] was synthesized from spectinomycin via catalytic hydrogenation of keto group, cbz protection of amines, and diacetonide protection diol. Deoxygenation at C-6 position in compound **14** was achieved efficiently by following Barton’s radical deoxygenation [23] method (Scheme 1). Thus, selective installation of imidazolyl thiocarbamate group on C-6-hydroxy group was carried out using 1,1′-thiocarbonyldimimidazole in the presence of catalytic amount of DMAP and treatment of thiocarbamate with tributyltin hydride and AIBN gave deoxygenated product **15** in good yields. An acetonide group was removed under 1 M HCl in methanol to give diol and selective oxidation of 4′-hydroxy group with IBX yielded cbz protected 6-deoxyspectinomycin **16**. Lastly, compound **16** was converted to 6-deoxyspectinomycin [20] **5** by removal cbz group under catalytic hydrogenation (Scheme 1). To achieve 6-deoxyspectinamide **6**, compound **16** underwent reductive amination reaction with ammonium nitrate and 2-picoline-borane complex to provide 4′*R*-amino-6-deoxyspectinomycin, which then coupled with 2-pyridyl acetic acid to give compound **17** [45, 46]. The newly generated stereochemistry on the 3′(*R*) position was assigned using coupling constant (*J*_3′4′ax_ = 4.4 Hz; *J*_3′4′eq_ = 2.3 Hz) [47] values between 3′ and 4′ hydrogens by synthesizing compound **16b** (data available in Supplementary Scheme 1, compound **16b**). Finally, catalytic hydrogenolysis of cbz product of **17** under Pd/C gave 6-deoxyspectinamide **6**.

**Scheme 2:**
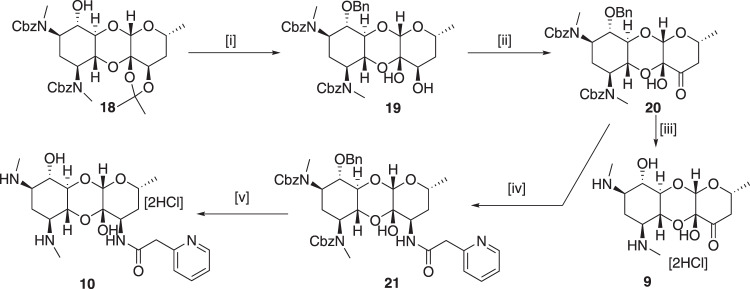
Synthesis of 2-deoxyspectinomycin **9** and 2-deoxyspectinamide **10**. Reagents and conditions: [i] (a) NaH, BnBr, THF, (b) 1 M HCl in MeOH, 65% over two steps; [ii] IBX, DMSO; 86% [iii] Pd/C-H_2_, MeOH, 80%; [iv] (a) NH_4_NO_3_/CH_3_COOH, 2-picoline-borane complex, MeOH, (b) 2-Pyridylacetic acid hydrochloride, HBTU, DMF, 34% over 2 steps; [v] Pd/C-H_2_, 1 M HCl in MeOH, 79%

Attempts to deoxygenation of C-2-hydroxy group using the Barton imidazolylthiocarbamate intermediate failed presumably due to the steric hindrance of C-2-hydroxy group, we consequently followed literature procedure to make 2-deoxyspectinomycin. The 2-deoxyderivative **18** was achieved from spectinomycin using the protocol of Foley et al. (Scheme 2) [21]. Benzyl protection of 6-hydroxy group followed by removal of acetonide group yielded diol **19**. Compound **9** was achieved by oxidation of compound **19** to give compound **20** and followed by cbz group deprotection. The 2-deoxyspectinamide **10** was synthesized from **20** via **21** following similar protocol [45] as used in **6**.

**Scheme 3:**
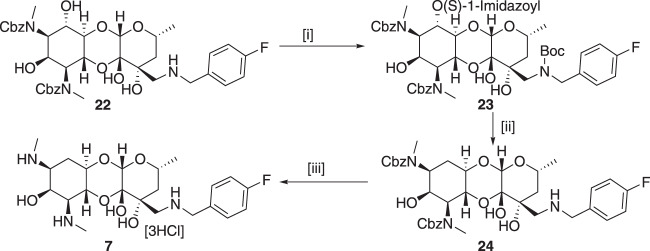
Synthesis of 6-deoxy-amSPC **7**. Reagents and conditions: [i] (a) (Boc)_2_O, Et_3_N, MeOH, (b) 1,1′-Thiocarbonyldiimidazole, DMAP, DCM, 71% overs two steps; [ii] (a) Bu_3_Sn-H/AIBN, Toluene, (b) TFA, DCM, 41% over two steps; [iii] Pd/C-H_2_, 1 M HCl in MeOH, 70%

**Scheme 4:**
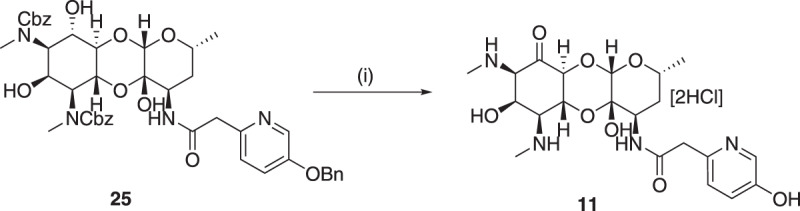
Synthesis of 6-dehydrospectinamide **11**. Reagents and conditions: [i] (a) pyridinium trifluoroacetate, dicyclohexyl carbodiimide, DMSO, 50 °C, (b) 10% Pd/C-H_2_, 0.1 M HCl in MeOH, 73%

6-Deoxy-amSPC **7** was synthesized from amSPC intermediate **22** [11]. First, the benzyl amine group in **22** [11] was protected as *tert*-butyl carbamate derivative and then the 6-imidazolylthiocarbamate derivative **23** was synthesized using 1,1′-thiocarbonyl diimidazole. Deoxygenation at C-6 position was then achieved efficiently by following Barton’s radical deoxygenation [23] method as used in compound **15** to give deoxygenated product **24** in moderate yield. Finally, deoxy compound **7** was achieved by removing *tert*-butoxy carbonyl group using trifluoroacetic acid and benzyloxy carbonyl group deprotection under hydrogenolysis condition (Scheme 3). The 6-*epi*-chloro compound **8** was synthesized following literature protocol [24]. The synthesis of 6-dehydrospectinamide **11** was accomplished from cbz protected **25** [25] via selective oxidation of C-6-hydroxy group by Pfitzner–Moffatt method and followed hydrogenolytic deprotection of cbz groups (Scheme 4).

### Antibacterial susceptibility and ribosomal activity of deoxy analogs

The antibacterial susceptibilities of the synthesized deoxy analogs were tested against a panel of Gram (+) and Gram (−) pathogens, plus *M. tuberculosis* (abbreviated in Table 1, full data available in Supplementary Table 1). All deoxyspectinomycin analogs lacked MIC activity in line with previous reports [20]. Deoxyspectinamides **6** and **10** and deoxy-amSPC **7** had some limited MIC activity against *M. tuberculosis* and *S. pneumoniae*, weaker than their corresponding parent spectinamide **2** and amSPC **3**, consistent with their reduced ribosomal binding affinities described subsequently.

**Table 1:** Antibacterial susceptibility (MIC, μg ml^−^^1^) and ribosomal affinity IC_50_ (μg ml^−1^) studies of synthesized compounds

Compounds	*M. tuberculosis* H37Rv MIC (µg ml^−1^)	*S. pneumoniae* R6 MIC (μg ml^−1^)	*E. coli* K-12 MIC (µg ml^−1^)	*E. coli* cellular accumulation (µM)	Ribosome translation inhibition IC_50_ (μg ml^−1^)
Spectinomycin (**1**)	50	12.5	25	1.1 ± 0.1	0.36
**2**	1.6	12.5	200	5.4 ± 0.8	0.89
**3**	6.25	3.13	50	40 + 1	0.74
**4**	1.6	12.5	100	5.9 + 0.8	0.84
**5**	>200	>200	>200	2.3 + 0.4	>60
**6**	50	25	>200	10.0 + 0.6	9.50
**7**	100	25	>200	47 + 1	23.3
**8**	>200	>200	>200	1.29 ± 0.06	54.7
**9**	>200	>200	>200	<0.4	>60
**10**	200	50	>200	5.6 ± 0.5	10.2
**11**	100	>200	>200	1.77 ± 0.06	34.7

The ability of the analogs to inhibit ribosomal translation was performed with *M. smegmatis* ribosomes in a cell-free translation assay [11] (Table 1). The 6- and 2-deoxyspectinomycins, **5** and **9**, show poor ribosomal binding inhibition (Ribo IC_50_ > 60 μg ml^−1^) compared to spectinomycin **1** (Ribo IC_50_ 0.36 μg ml^−1^). The molecular docking of **5** and **9** suggests that the hydrogen bonding interactions from the 6- and 2-hydroxy groups are crucial for ribosomal binding inhibition (Fig. S2A, B). Substitution of the hydroxy group at the 6-position on spectinomycin was tested to determine if the loss in activity could be rescued while avoiding AME activity. However, addition of a chlorine at the 6-position (**8**) maintained poor activity (Table 1) and disrupted the majority of hydrogen bonding interactions involved with spectinomycin binding (Fig. S2C). Therefore, the hydroxy group at the 6-position of spectinomycin forms hydrogen bonding interactions that are crucial to maintain ribosomal inhibition.

The 6-deoxyspectinamide **6** showed improved ribosomal inhibition compared to **5**, (Ribo IC_50_ = 9.50 μg ml^−1^) suggesting the aryl side chain could partially rescue inhibitor activity. The aryl side chain extends from the spectinomycin binding site and interacts directly with the RpsE protein loop through VDW interactions (Fig. S3). Similarly, 6-deoxy-amSPC **7** had improved ribosomal inhibition (Ribo IC_50_ = 23.3 μg ml^−1^) from the contribution of the aryl side chain (Fig. S4). This feature may be used in part to compensate for the loss of hydrogen bonding interaction with the spectinomycin core and the bacterial ribosome, a phenomenon that could not be observed in **5** as it lacks the aryl side chain. 2-Deoxyspectinamide **10** also showed improved ribosomal inhibition (Ribo IC_50_ = 10.2 μg ml^−1^) compared to the 2-deoxyspectinomycin **9** (Ribo IC_50_ > 60 μg ml^−1^). From the molecular docking of **10**, the aryl side chain is also shown to gain interactions with the RpsE protein backbone, providing further evidence that aryl side chain rescues ribosomal binding (Fig. S3).

In the case of the 6-dehydrospectinamide **11**, we observed an increase in ribosomal binding affinity compared to other 6-deoxy analogs (Ribo IC_50_ = 34.7 μg ml^−1^). The molecular docking studies of the parent compound, **4**, and the **11** analog reveal the carbonyl at the 6-position docked with a similar predicted affinity (Fig. S5). The exact molecular entity of **11** is not known in solution state at this stage as it likely exists in equilibrium with its hydrated form as indicated by its ^13^C NMR (Fig. 3). Therefore, we also docked the **12** (*gem-*diol-**11**) (Fig. S5). This analog exhibited two hydrogen bond interactions with G1064 and G1193, but loses the hydrogen bonding interaction with the backbone of Arg60 and has a weaker docking score compared to **4** and **11** (Fig. S5). These results point to reason for the weak binding (Ribo IC_50_ = 34.7 μg ml^−1^) for **11** compared to **4** (Ribo IC_50_ = 0.84 μg ml^−^^1^) is due to **12** (*gem*-diol-**11**) being the dominant binding isoform.

### Cellular accumulation

**Fig. 5:**
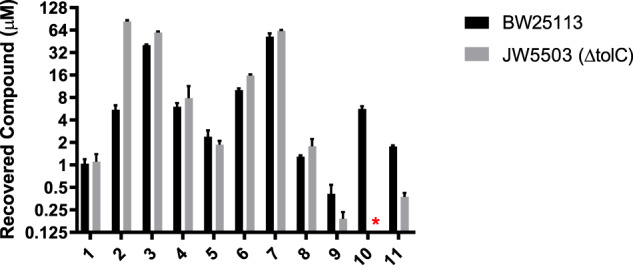
Whole-cell accumulation assay tested per compound in wild-type *E. coli*. (BW25113) and Δtolc (JW5503) strains. *Not determined

To examine if altered cellular accumulation contributes to reduced MIC activity of deoxyspectinomycin analogs, the concentration of all analogs within *E. coli* cells was measured and compared to their corresponding spectinomycin family parent (Fig. 5 and Table S3) [43]. These experiments indicated a substantial difference in the accumulation between the different families, spectinomycins (**1**, **5**, **8**, **9**; range <0.4 to 1.2 μM), spectinamides (**2**, **4**, **6**, **10**; range <5.4 to 10 μM), and amSPCs (**3**, **7**; 40 and 47 μM, respectively). When examining matched pairs within families (**1** vs **5** and **9**; **2** vs **6** and **10**; **3** vs **7**), deoxygenation led to a slight increase in accumulation, with the most pronounced effect for 6-deoxy analogs. The increased accumulation is, however, modest and unlikely to impact MIC levels. The pattern of uptake was generally mirrored in the *E. coli* ΔtolC strain. However, these data suggest that the early spectinamide, **2**, is subjected to efflux by tolC. The depletion of the efflux pump results in a 15-fold increase in intracellular accumulation, which matches the trends in susceptibility where knocking out the efflux pump reduces the MIC of **2** from 200 to 50 μg ml^−1^. Overall, these results indicate that altered accumulation does not affect the antimicrobial potency among the deoxyspectinomycin analogs.

### Molecular dynamics simulations

**Fig. 6:**
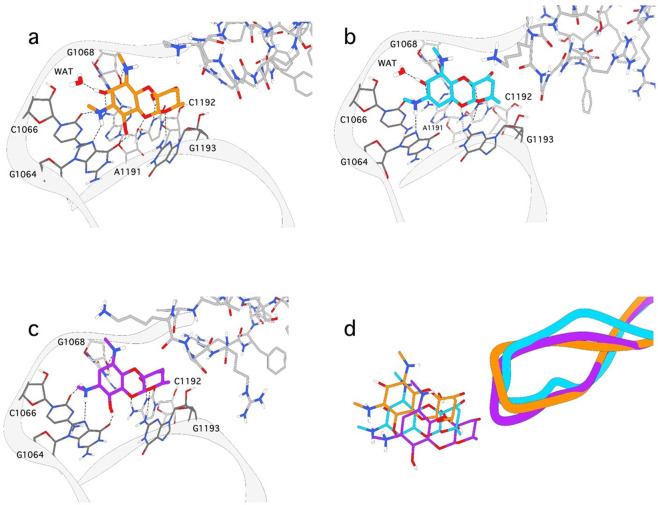
Snapshot from 20 ns molecular dynamic simulation of spectinomycin analogs. The binding mode of **a** spectinomycin, **b 5**, and **c 9** from 20 ns MD simulations. The protein is shown as a gray ribbon with nucleotides and waters interacting with the ligand are labeled. The hydrogen bonding interactions are depicted as dashed lines. **d** An overlay of spectinomycin (orange), **5** (cyan), and **9** (purple) from the 20 ns MD simulations

**Fig. 7:**
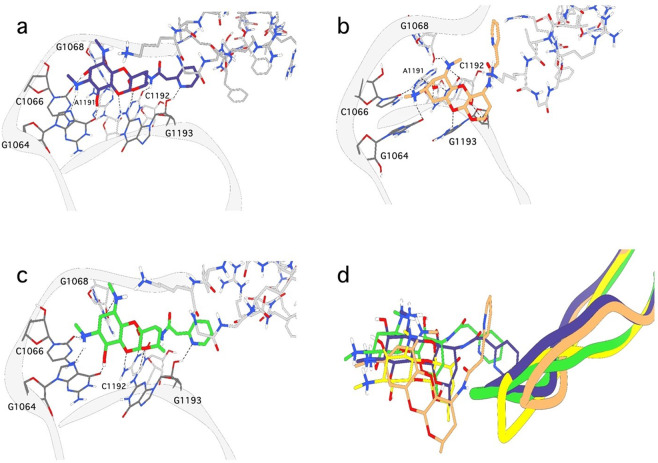
Modeled binding modes of spectinamide analogs from 20 ns molecular dynamic simulation in AMBER18. The binding mode of **a 2**, **b 6**, and **c 10** from 20 ns MD simulations. The protein is rendered as a gray ribbon with nucleotides and waters interacting with the ligand shown as sticks. The dashed lines represent hydrogen bonding interactions. **d** An overlay of spectinomycin (yellow), **2** (purple), **6** (orange), and **10** (green) from the 20 ns MD simulations

Spectinomycin binding is mediated through a complex pattern of hydrogen bond interactions between the rRNA nucleotides and the ligand [26]. A better understanding of the contributions of the 2- and 6-hydroxy group positions on the spectinomycin scaffold is crucial for the development of novel analogs that overcome inactivation by AMEs. We chose to examine these interactions using molecular dynamics simulations, rather than the rigid body docking used earlier in this paper. These are computationally intense experiments that account for dynamically changing interactions between the ligand, rRNA nucleotides, RpsE loop, and associated water molecules. Removing or modifying the 2- and 6-hydroxy groups on spectinomycin and analogs had large effect on the binding and antibacterial activity (Table 1). To examine the molecular basis of these effects, the docked conformations of spectinomycin **2**, **5**, **6**, **9**, and **10** analogs were subjected to a 20 ns molecular dynamic simulation (Figs. 6 and 7). The docking pose of spectinomycin into the *M. tuberculosis* rRNA/RpsE complex homology model was compared to the *E. coli* X-ray crystal structure (PDB ID: 2QOU [26]) to ensure the binding mode was not altered between species. The ligand RMSD between the two structures was 0.65 Å (data not shown). The final spectinomycin binding mode following a 20 ns MD simulation is shown (Fig. 6). Like the X-ray crystal structure, the nucleotides in the spectinomycin binding pocket participate in several key hydrogen bonding interactions. The 2-hydroxy group coordinates with a water molecule and interacts with A1191, while the 6-hydroxy group has one binding interaction with G1064.

In the simulation, 6-deoxyspectinomycin **5** lost the 6-hydroxy group-mediated hydrogen bonding interaction with the G1064 nucleotide (Fig. 6b) and resulted in a 1.65 Å RMSD shift in the pocket compared to spectinomycin (Fig. 6d). The free energy of binding for spectinomycin and analogs was calculated using MM/GBSA in Amber18 [40] (Table 2). The MM/GBSA method calculates binding free energies using molecular mechanics at low computational cost [40, 48]. Furthermore, GBSA is accurate and robust in ranking binding affinities for structure-based drug discovery. Removal of the 6-hydroxyl group resulted in a decrease of 8.6 kcal mol^−1^ in binding energy compared to spectinomycin and is reflective of the poor IC_50_. Removal of the 2-hydroxy in **9** results in the loss of two hydrogen bonding interactions in the pocket, i.e., a water molecule and the A1191 nucleotide (Fig. 6c). Despite the remaining hydrogen bonding partners, **9** produces a larger shift in the pocket with a RMSD of 2.81 Å (Fig. 6d). This shift results in a 6.3 kcal mol^−1^ decrease in the binding energy compared to spectinomycin (Table 2). While both analogs had poor binding affinities, loss of the 2-hydroxy group was more favorable than the 6-hydroxy group for the spectinomycin core.

**Table 2:** Binding free energy onto the Mycobacterial RNA/RpsE complex determined using the MM/GBSA method in Amber18

Compound	*E*_VDW_^a^ (kcal^−1^ mol^−^^1^)	*E*_nonpolar_^a^ (kcal^−1^ mol^−1^)	Δ*G*_total_^a^ (kcal^−1^ mol^−1^)	Ribosomal IC_50_^b^ (μg ml^−1^)
Spectinomycin	−38.9 ± 2.70	−5.41 ± 0.05	−78.5 ± 2.32	0.36
**5**	−36.9 ± 2.60	−5.21 ± 0.05	−69.9 ± 2.92	>60
**9**	−39.3 ± 2.55	−5.34 ± 0.05	−72.2 ± 2.21	>60
**2**	−57.2 ± 2.98	−7.29 ± 0.06	−97.6 ± 2.78	0.89
**6**	−49.8 ± 2.76	−6.86 ± 0.05	−81.6 ± 2.49	9.50
**10**	−56.3 ± 2.91	−7.34 ± 0.06	−92.3 ± 2.27	10.2

^a^Values represent an average over the 1000 MD frames ± standard deviation

^b^Tested with *M. smegmatis*

Interactions driving spectinomycin binding are also observed in the spectinamide **2**-bound complex, with the addition of a hydrogen bond between the aryl chain on spectinamide and G1193 (Fig. 7a). The aryl chain also gains VDW interactions on the RpsE protein loop. Binding of **2** did not perturb the spectinomycin binding pocket, with a RMSD value of 0.75 Å (Fig. 7d). However, binding of **6**, the corresponding 6-deoxy analog, shifted 2.47 Å from **2** in the pocket and reduced the calculated Δ*G* of binding by 16.0 kcal mol^−1^ (Table 2). Despite the loss of one hydrogen bond interaction with G1064, **6** maintains VDW interaction RpsE protein loop (Fig. 7b), improving the IC_50_ compared to the 6-deoxyspectinomycin analog **5**. Additionally, **6** gains a hydrogen bonding interaction between G1193 phosphate backbone and the spectinomycin scaffold because of the large shift in the binding pocket (Fig. 7b). In contrast, the 2-deoxyspectinamide analog **10** remains in the spectinomycin binding pocket with a RMSD value of 1.28 Å compared to **2** (Fig. 7d). Losing the A1191 hydrogen bond is alleviated through interactions from the aryl chain to G1193 and VDW interactions with Val56 on the RpsE protein loop (Fig. 7c). Binding of **10** reduced the Δ*G* of binding by 5.3 kcal mol^−1^ compared to **2**, suggesting the loss of the 2-hydroxy group is more favorable than the 6-hydroxy group as observed with deoxyspectinomycin analogs. However, despite loss of the hydroxy groups, both deoxyspectinamide analogs recover binding interactions and rescue activity via additional protein–ligand interactions on the RpsE protein loop.

**Fig. 8:**
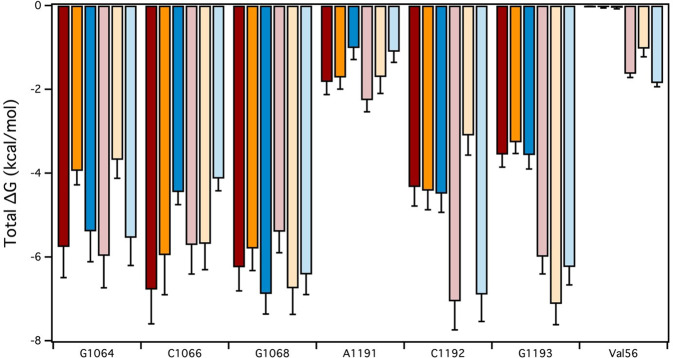
Decomposition of free energy of binding in the spectinomycin binding pocket. The total Δ*G* of binding for each nucleotide interacting with spectinomycin **1** (dark red), **5** (dark orange), **9** (dark blue), **2** (light red), **6** (light orange), and **10** (light blue) in the binding pocket. The Δ*G* is an average over the 20 ns MD ensemble and the error bars represent the standard deviation

The calculated Δ*G* of binding for the analogs correlates to the measured ribosomal IC_50_ data; however, the energetic contribution for each nucleotide is unknown from this analysis. Therefore, the free energy decomposition [40] of each nucleotide and amino acid in the binding pocket was compared between the analogs (Fig. 8). From the spectinomycin MD simulation, G1064, C1066, and G1068 have the strongest interaction in the binding pocket, with A1191, C1192, and G1193 being the weakest (Fig. 8 and Table S2). When the 6-hydroxy group is removed, the hydrogen bonding interaction from G1064 is lost and results in a 1.8 kcal mol^−1^ decrease in free energy (Fig. 8). The loss of the 6-hydroxy group indirectly effected nucleotide binding interactions with **5**, 0.82 kcal mol^−1^ loss for C1066 and 0.45 kcal mol^−1^ loss for G1068. In contrast, the removal of the 2-hydroxy group did not produce these large changes in the free energy of binding (Fig. 8). Despite the loss of A1191 hydrogen bonding interactions, the C1066 had the largest loss in free energy of 2.33 kcal mol^−1^. This trend was observed for the deoxyspectinamide analogs compared to the parent **2** compound. The 6-deoxy **6** analog had a decrease in G1064 free energy by 2.29 kcal mol^−1^, as the hydrogen bond interaction is lost as observed for **5**. Additionally, the binding free energy for C1192 decreased, 3.96 kcal mol^−1^, which was not observed for **5**. This change in C1192 binding energy may be a result of the large shift in the binding pocket. The aryl side chain increased binding interactions with G1193, 1.13 kcal mol^−1^. The 2-deoxy analog **10** maintained similar binding free energies compared to the parent compound **2**. This is not surprising, as **10** did not perturb the binding pocket, RMSD value of 1.28 Å. The loss of the A1191 binding interaction in the pocket resulted in a 1.16 kcal mol^−1^ decrease in energy, as well as indirect effect on C1066, 1.58 kcal mol^−1^. The aryl chains in the spectinamide series did increase binding interactions through the RpsE protein loop, as observed in the increase of Val56 free energy (Fig. 8).

From the decomposition of the free energy of binding, loss of the 2-hydroxy group in **9** and **10** did not greatly perturb the binding free energy of the nucleotides in the pocket except for a decrease in C1066 and A1191 (Fig. 8). However, loss of the 6-hydroxy group in **6** greatly impacted the free energy landscape of the binding pocket. Binding free energies decreased for G1064, A1191, and C1192 and increased with G1068 and G1193 (Fig. 8). This increase in energy is a result of shifting the spectinamide out of the pocket to maintain contacts between aryl side chain and the RpsE protein. This compensatory mechanism shown for both **6** and **10** suggests that the aryl side chain on these analogs promote favorable binding interactions, i.e., Val56 (Fig. 8). Structure-based drug discovery of the aryl chain on deoxyspectinamides to increase protein–ligand interactions may be a viable method to overcome the energetic penalties for removing the 2- or 6-hydroxy groups to avoid AME activity.

## Conclusions

Aminoglycoside resistance mediated by AME activity remains a major hurdle in the field of antibiotic drug discovery. This study sought to determine how removal or modification of the AME targeted motifs in spectinomycin, and the related spectinamides and amSPCs, affected ribosomal potency, antibacterial activity, and cellular accumulation. We used an efficient method for the deoxygenation of spectinomycin enabling the synthesis of deoxyspectinamides and amSPCs. This method involves formation of imidazolylthiocarbamate and followed by deoxygenation reaction with tributyltin hydride, as used in classical “Barton-McCombie” deoxygenation method. The deoxyspectinomycin analogs had poor antibacterial activity and ribosomal binding affinity, as did the 6-dehydrospectinamide **11** and the *epi*-chloro spectinomycin **8** also synthesized as comparators. Molecular dynamic simulations followed by energy decomposition of the individual nucleotides involved in spectinomycin binding revealed the 6-hydroxy group has a larger effect on binding compared to the 2-hydroxy group consistent with in vitro protein translational inhibition data. These results experimentally validate our modeling approach, which allow for key binding site residues to be targeted in the design of future spectinomycin analogs.

Other important spectinomycin binding/structure–activity information can be gleaned from examining the testing data in this study, including an evaluation of the contribution the aryl side chains make to spectinamide and amSPC binding. In this case, the loss of binding affinity caused by deoxygenation of the spectinomycin ring affinity is demonstrated to be partially rescued by the additional interactions between the RpsE protein and the aryl side chains extending from the deoxyspectinamide and deoxy-amSPC analogs. Deoxygenation decreases the hydrophilicity of the spectinomycins, a goal of our program, as more lipophilic analogs are expected to have higher permeability in mammalian and bacteria cells. However, deoxygenation of the analogs in this study led to only small increases in cellular accumulation and was not found to affect the antimicrobial potency.

This study builds the on the knowledge of the SAR of spectinomycin analogs and is being used to aid the design of next-generation spectinomycins. Analogs that maintain a non-AME modifiable hydrogen bond donor at the 6-position to interact with G1064 in combination with aryl side chains modifications are being explored in subsequent studies. Such analogs may be successful new class of antibiotics targeting the helix-34 ribosomal binding site, overcoming AME resistance, if their overall binding affinities can be increased and their cellular permeability optimized.

## Supplementary information

supplementary data
