# Supplementary material for: Synthesis, antibacterial action, and ribosome inhibition of deoxyspectinomycins
Source: J Antibiot (Tokyo). 2021 Jan 27;74(6):381–96. doi: 10.1038/s41429-021-00408-3 (PMC8154590; doi:10.1038/s41429-021-00408-3)
Supplement: Supplementary file 1 — supplementary data [file 41429_2021_408_MOESM1_ESM.docx]

**Supplementary information for**

**Synthesis, Antibacterial Action and Ribosome Inhibition of Deoxyspectinomycins**

Suresh Dharuman^1^, Laura A. Wilt^1^, Jiuyu Liu^1^, Stephanie M. Reeve^1^, Carl W. Thompson^1^, John M. Elmore^1^, Dimitri Shcherbakov,^2^ Robin B. Lee^1^, Erik C. Böttger^2^ and Richard E. Lee^1^*

^1^Department of Chemical Biology and Therapeutics, St. Jude Children’s Research Hospital, Memphis, Tennessee

^2^Institut für Medizinische Mikrobiologie, Universität Zürich, 8006 Zürich, Switzerland

| **Supplementary** Synthesis and Characterization of compounds **15 - 24** | **S2-S9** |
| --- | --- |
| **Supplementary** Table 1 | **S10** |
| **Supplementary** Table 2 | **S11-S12** |
| **Supplementary** Table 3 | **S13** |
| **Supplementary** Figure 1 | **S14** |
| **Supplementary** Figure 2 | **S15** |
| **Supplementary** Figure 3 | **S16** |
| **Supplementary** Figure 4  **Supplementary** Figure 5 | **S17**  **S18** |
| **Supplementary** References  **Supplementary** Copies of ^1^H and ^13^C NMR Spectra of Compounds **5** - **10** | **S19**  **S20-S29** |

**Synthesis and Characterization**

1, 3-*N*, *N'* –bis(benzyloxycarbonyl)-6-deoxy-3'-dihydro-2',3'-*O*-isopropylidenespectinomycin (**15**)

1,1’-thiocarbonyldiimidazole (7.93 g, 44.50 mmol) and DMAP (0.247 g, 2.023 mmol) was added to a stirred solution of 1, 3-*N,N-*bis(benzyloxycarbonyl)-2',3'-*O*-isopropylidenedihydrospectinomycin **14**^1^ (13.0 g, 20.23 mmol) in dry CH_2_Cl_2_ (300 mL) at room temperature. The reaction mixture was stirred at room temperature for 12 hours before it was evaporated to give the crude product. The crude product was purified through short silica-gel column chromatography to give imidazoyl thiocarbamate intermediate. Bu_3_Sn-H (5.5 mL, 20.46 mmol) and AIBN (7.31 mL, 1.461 mmol; 0.2 M soln. in toluene) was added to a solution of imidazoyl thiocarbamate (11.0 g, 14.61 mmol) dissolved in toluene (400 mL) at room temperature and heated at reflux for 15 minutes. The reaction mixture was evaporated under high vacuum to give a crude product and purified on silica-gel column chromatography (Eluents: 40% EtOAc in hexane) to afford pure compound **15** (7.0 g, 55% over two steps) as a white solid. ^1^H NMR (500 MHz, CD_3_OD) δ 7.48 – 7.12 (m, 10H), 5.35 – 4.90 (m, 4H), 4.63 (s, 1H), 4.28 (t, *J* = 10.4 Hz, 1H), 4.20 (s, 1H), 4.14 – 4.10 (m, 2H), 4.03 – 3.94 (m, 2H), 3.89 – 3.79 (m, 1H), 3.11 (s, 3H), 2.99 (s, 3H), 2.14 (q, *J* = 12.1 Hz, 1H), 1.93 (dd, *J* = 15.2, 7.2 Hz, 1H), 1.84 – 1.61 (m, 2H), 1.47 (s, 3H), 1.43 (s, 3H), 1.24 (d, *J* = 6.4 Hz, 3H); ^13^C NMR (125 MHz, CD_3_OD) δ 157.3, 156.9, 136.8, 136.7, 128.2, 128.1, 127.7, 127.6, 127.5, 109.3, 97.5, 96.1, 77.4, 70.0, 69.7, 68.9, 68.1, 66.9, 57.0, 53.4, 33.3, 33.2, 30.2, 30.1, 26.6, 24.6, 19.7; HRMS (ESI) m/z calcd for C_33_H_43_N_2_O_10_ [M+H]^+^, 627.2918; found, 627.2916.

1, 3-*N*, *N* –(bis-benzyloxycarbonyl)-6-deoxyspectinomycin (**16**)

A solution of 1, 3-*N*, *N'* –(bis-benzyloxycarbonyl)-6-deoxy-3'-dihydro-2',3'-(di-*O*-isopropylidene)-6-deoxydihydrospectinomycin **15** (1.0 g, 1.60 mmol) dissolved in 1 M HCl in MeOH (15 mL) and 1 M HCl in H_2_O (15 mL) was stirred at 70 ^o^C for 0.5 hours. The reaction mixture was evaporated under high vacuum to give a crude product. The crude product was dissolved in EtOAc (20 mL) and washed with saturated NaHCO_3_ solution (10 mL). The organic layer was dried over Na_2_SO_4,_ and concentrated to give diol. IBX (0.44 g, 1.58 mmol) was added to the solution of diol (0.93 g, 1.58 mmol) in DMSO (10 mL) at room temperature and stirred for 14 hours. The reaction was quenched with water (10 mL), extracted with EtOAc (2 x 20 mL), dried over Na_2_SO_4_, and concentrated. The crude product was purified on silica-gel column chromatography (Eluents: 50% EtOAc in hexane) to afford pure compound **16** (0.80 g, 85% over two steps) as a white solid.^1^H NMR (500 MHz, CD_3_OD) δ 7.53 – 7.05 (m, 10H), 5.36 – 4.97 (m, 4H), 4.66 (s, 1H), 4.51 (t, *J* = 10.6 Hz, 1H), 4.38 – 4.28 (m, 1H), 4.26 – 4.01 (m, 2H), 3.78 (dt, *J* = 12.2, 6.1 Hz, 1H), 3.07 (s, 3H), 3.02 (s, 3H), 2.95 – 2.82 (m, 1H), 2.71 (dt, *J* = 41.2, 13.0 Hz, 1H), 2.39 (dd, *J* = 14.1, 5.6 Hz, 1H), 2.21 (qd, *J* = 12.3, 4.3 Hz, 1H), 1.81 (s, 1H), 1.37 (d, *J* = 6.6 Hz, 3H); ^13^C NMR (125 MHz, CD_3_OD) δ 171.6, 157.4, 156.8, 136.9, 136.8, 128.2, 128.1, 127.7, 127.6, 127.5, 127.4, 127.3, 96.9, 91.7, 68.9, 67.7, 67.0, 66.8, 66.7, 60.2, 56.8, 53.6, 45.0, 44.9, 30.4, 20.3, 19.5, 13.1; HRMS (ESI) m/z calcd for C_30_H_37_N_2_O_10_ [M+H]^+^, 585.2448; found, 585.2435.

3'(*R*)-Amino-3'-deoxy-6-deoxyspectinomycin trihydrochloride(**16b**)

Ammonium nitrate (0.41 g, 5.13 mmol) and 2-picoline borane complex (0.03 g, 0.31 mmol) was added to a solution of ketone **16** (0.30 g, 0.51 mmol) in MeOH (3.0 mL) and AcOH (0.5 mL) at room temperature and stirred for 30 minutes. The reaction mixture was evaporated and diluted with saturated NaHCO_3_ solution, the compound was extracted with EtOAc (2 x 20 mL), dried over Na_2_SO_4_, and concentrated to give crude amine. The crude amine was purified on silica-gel column chromatography to yield pure amine **16a**. ^1^H NMR (500 MHz, CD_3_OD) δ 7.45 – 7.17 (m, 10H), 5.27 – 4.92 (m, 4H), 4.82 (s, 1H), 4.44 (t, *J* = 10.5 Hz, 1H), 4.22 – 3.94 (m, 5H), 3.06 (s, 3H), 3.05 (s, 3H), 3.01 – 2.99 (m, 1H), 2.18 (qd, *J* = 12.0, 4.5 Hz, 1H), 1.87 – 1.65 (m, 2H), 1.57 (d, *J* = 13.9 Hz, 1H), 1.21 (d, *J* = 7.0, 3H). Pd/C (15 mg) was added to amine **16a** (0.15 g, 0.25 mmoL) dissolved in 1 M HCl in MeOH (3 mL) and the reaction mixture was stirred for 4 hours under H_2_ atmospheric pressure. Excess catalyst was filtered off and washed with acetone (5 mL) to produce the pure product **16b** (0.05 g, 23% over 2 steps) as a white solid. ^1^H NMR (500 MHz, D_2_O) δ 5.02 (s, 1H), 4.66 (d, *J* = 3.1 Hz, 1H), 4.31 (t, *J* = 10.4 Hz, 1H), 4.20 – 4.10 (m, 2H), 3.67 (dd, *J* = 4.4, 2.3 Hz, 1H), 3.52 – 3.43 (m, 2H), 2.83 (s, 3H), 2.80 (s, 3H), 2.29 – 2.24 (m, 1H), 2.09 (ddd, *J* = 16.0, 11.9, 4.4 Hz, 1H), 2.00 – 1.86 (m, 2H), 1.30 (d, *J* = 6.0 Hz, 3H); ^13^C NMR (125 MHz, D_2_O) δ 92.6, 88.7, 68.1, 66.9, 65.2, 61.6, 58.7, 55.8, 52.8, 32.0, 30.8, 30.6, 25.3, 19.6; HRMS (ESI) m/z calcd for C_14_H_28_N_3_O_5_ [M+H]^+^, 318.2029; found, 318.2023. The stereochemistry on 3'(*R*) position was confirmed by calculating coupling constant of 3' hydrogen with 4' hydrogens (*J*_3',4'ax_ = 4.4 Hz, *J*_3',4'eq_=2.3 Hz)^2^.

**Scheme 1. Synthesis of compound 17.**

*1, 3-N,N'*-(bis-Benzyloxycarbonyl)-6-deoxy-3'-deoxy-3'(*R*)-dihydro)-[(pyridin-2-yl)acetylamino]spectinomycin (**17**)

Ammonium nitrate (0.068 g, 0.85 mmol) and 2-picoline borane complex (0.018 g, 0.17 mmol) was added to a solution of ketone **16** (0.050 g, 0.086 mmol) dissolved in MeOH (2 mL) and AcOH (0.025 mL) at room temperature and stirred for 30 minutes. The reaction mixture was evaporated and diluted with saturated NaHCO_3_ solution and the compound was extracted with EtOAc (4 x 20 mL), dried over Na_2_SO_4_, and concentrated to give amine **16a**. The amine **16a** (0.025 g, 0.043 mmoL) was dissolved in DMF (1.0 mL) and treated with 2-pyridylacetic acid hydrochloride (0.015 g, 0.085 mmol), HBTU (0.016 g, 0.043 mmol), and Et_3_N (0.018 mL, 0.128 mmol) at room temperature. After 15 minutes, the reactions was quenched with water (5 mL), extracted with EtOAc (3 x 10 mL), dried over Na_2_SO_4_, and concentrated. The crude product was purified on silica-gel column chromatography (Eluents: 0-5% MeOH in CH_2_Cl_2_) to give pure product **17** (0.015 g, 25% over two steps) as a colorless oil. ^1^H NMR (500 MHz, CD_3_OD) δ 8.50 (dt, *J* = 4.9, 1.5 Hz, 1H), 7.81 (td, *J* = 7.7, 1.9 Hz, 1H), 7.49 – 7.20 (m, 12H), 5.28 – 5.01 (m, 4H), 4.87 (s, 1H), 4.62 (br s, 1H), 4.53 (ddd, *J* = 11.8, 9.3, 2.7 Hz, 1H), 4.22 – 3.99 (m, 4H), 3.98 – 3.89 (m, 1H), 3.87 – 3.80 (m, 2H), 3.09 (s, 3H), 3.01 (s, 3H), 2.27 – 2.12 (m, 1H), 1.87 – 1.66 (m, 3H), 1.22 (d, *J* = 6.2 Hz, 3H); ^13^C NMR (125 MHz, CD_3_OD) δ 171.1, 157.5, 155.5, 148.4, 137.4, 128.2, 128.2, 127.7, 127.6, 127.5, 127.5, 127.5, 127.2, 124.4, 122.2, 94.1, 94.1, 90.1, 90.1, 68.5, 66.9, 66.8, 66.7, 56.8, 53.0, 43.8, 34.9, 34.8, 30.3, 19.8; HRMS (ESI) m/z calcd for C_37_H_45_N_4_O_10_ [M+H]^+^, 705.3136; found, 705.3127.

6-*O*-Benzyl-1, 3-*N*, *N'*–(bis-benzyloxycarbonyl)-2-deoxy-3'(*R*)-dihydrospectinomycin (**19**)

NaH (0.077 g, 3.19 mmol) and benzyl bromide (0.190 mL, 1.59 mmol) was added to a stirred solution of alcohol **18**^3^ (1.0 g, 1.59 mmol) in THF (20 ml) at 0 ^o^C and the reaction mixture was stirred for 1 hour at room temperature. After 1 hour, the reaction was quenched with water (10 mL), extracted with EtOAc (2 X 50 mL), dried over Na_2_SO_4_, and concentrated. After short silica-gel column chromatography, the product was dissolved in 1 M HCl water (20 mL) and MeOH (20 mL) and heated to 90 ^o^C for 30 minutes. The reaction was evaporated and dissolved in EtOAc, washed with NaHCO_3_ solution, dried over Na_2_SO_4_, and concentrated. The crude product was purified on silica-gel column chromatography (Eluents: 50% EtOAc in hexane) to give pure diol **19** (0.70 g, 65% over 2 steps) as a white solid. ^1^H NMR (500 MHz, CD_3_OD; 1: 0.5 ration of rotamers) δ 7.49 – 7.20 (m, 15H, both rotamers), 5.37 – 5.17 (m, 4H, minor rotamer), 5.12 – 5.04 (m, 4H, major rotamer), 4.90 (s, 1H, both rotamers), 4.87 – 4.85 (m, 2H, major rotamer), 4.61 – 4.54 (m, 2H, minor rotamer), 4.34 – 3.98 (m, 8H, both rotamers), 3.63 – 3.61 (m, 2H, both rotamers) 2.87 (s, 3H, both rotamers) 2.67 (s, 3H, both rotamers), 1.79 (t, *J* = 12.6 Hz, 1H, both rotamers), 1.73 – 1.56 (m, 3H, both rotamers), 1.25 (d, *J* = 6.1 Hz, 3H, major rotamer), 1.19 (d, *J* = 6.1 Hz, 3H, minor rotamer); ^13^C NMR (125 MHz, CD_3_OD) δ 157.0, 138.8, 138.7, 138.6, 138.5, 136.9, 136.8, 128.2, 128.1, 127.9, 127.8, 127.8, 127.7, 127.6, 127.6, 127.5, 127.3, 127.1, 93.5, 89.9, 73.4, 70.4, 70.3, 70.3, 67.3, 67.0, 66.9, 66.8, 66.0, 37.1, 36.9, 28.0, 19.9, 19.8, 13.6; HRMS (ESI) m/z calcd for C_37_H_45_N_2_O_10_ [M+H]^+^, 677.3074; found, 677.3070.

6-*O*-Benzyl-1, 3-*N*, *N'*–(bis-benzyloxycarbonyl)-2-deoxyspectinomycin (**20**)

IBX (0.58 g, 2.07 mmol) was added to diol **19** (0.70 g, 1.03 mmol) in DMSO (8 mL) at room temperature and stirred for 12 hours. The reaction was quenched with saturated Na_2_S_2_O_3_ solution (10 mL), extracted with EtOAc (2 x 10 mL), dried over Na_2_SO_4_, and concentrated to give crude product, which was purified on silica-gel column chromatography (Eluents: 50% EtOAc in hexane) to afforded pure compound **20** (0.60 g, 86%) as a white solid. ^1^H NMR (500 MHz, CD_3_OD; 1:0.5 mixture of rotamers) δ 7.47 – 7.05 (m, 15H, both rotamers), 5.37 – 4.95 (m, 4H, both rotamers), 4.94 – 4.89 (s, 2H, both rotamers), 4.77 – 4.74 (m, 1H, both rotamers), 4.68 – 4.43 (m, 2H, both rotamers), 4.48 – 3.88 (m, 4H, both romaters), 3.85 – 3.59 (m, 2H, both rotamers), 2.91 (s, 3H, both rotamers), 2.66 (s, 3H, both rotamers), 2.60 – 2.30 (m, 3H, minor rotamer), 2.41 – 2.31 (m, 1H, minor rotamer) 2.13 – 1.82 (m, 1H, major rotamer), 1.72 – 1.57 (m, 3H, major rotamer), 1.39 (m, 3H, major rotamer), 1.26 (m, 3H, minor rotamer); ^13^C NMR (125 MHz, CD_3_OD) δ 157.0, 156.8, 138.8, 138.7, 138.6, 137.0, 136.8, 128.2, 128.2, 128.1, 128.0, 127.9, 127.8, 127.7, 127.6, 127.5, 127.3, 127.2, 127.1, 96.5, 96.3, 94.4, 94.2, 91.7, 91.3, 73.8, 73.7, 73.3, 73.1, 67.8, 67.7, 67.5, 67.1, 67.0, 66.9, 66.8, 45.1, 44.9, 41.1, 36.4, 36.2, 28.1, 28.0, 20.3, 19.7, 13.7; HRMS (ESI) m/z calcd for C_37_H_43_N_2_O_10_ [M+H]^+^, 675.2918; found, 675.2896.

6-*O*-Benzyl-1, 3-*N,N'*-(bis-benzyloxycarbonyl)-2-deoxy-3'-deoxy-3'(*R*)-dihydro-[(pyridin-2-yl)acetylamino]spectinomycin (**21**)

Compound **21** (0.040 g, 34% over 2 steps) was synthesized analogously as **17** from **20**, as a white solid. ^1^H NMR (500 MHz, CD_3_OD) δ 8.46 – 8.32 (m, 1H), 7.70 (td, *J* = 7.7, 1.8 Hz, 1H), 7.41 – 7.05 (m, 17H), 5.29 – 4.89 (m, 4H), 4.81 (s, 1H), 4.53 – 4.39 (m, 1H), 4.33 – 3.86 (m, 6H), 3.89 – 3.62 (m, 3H), 2.76 (s, 3H), 2.68 – 2.64 (m, 1H), 2.55 (s, 3H), 1.73 – 1.50 (m, 4H), 1.14 (d, *J* = 6.8 Hz, 3H); ^13^C NMR (125 MHz, CD_3_OD) δ 171.1, 157.0, 155.5, 148.4, 137.5, 136.8, 128.2, 128.2, 128.2, 127.9, 127.7, 127.6, 127.5, 127.1, 124.4, 122.2, 93.6, 89.7, 68.9, 67.5, 67.0, 66.8, 52.9, 43.8, 40.3, 35.0, 34.8, 29.4, 29.3, 28.5, 19.9, 19.8, 12.9, 11.7; HRMS (ESI) m/z calcd for C_44_H_51_N_4_O_10_ [M+H]^+^, 795.3605; found, 795.3622.

*1, 3-N,N*-(bis-Benzyloxycarbonyl)-6-*O*-(imidazoylthiocarbonyl)-3’(*R*)-3’-[(4-fluoro)benzylaminomethyl)]dihydrospectinomycin (**23**)

di-*tert*-butyl dicarbonate (0.059 g, 0.27 mmol) and Et_3_N (0.057 mL, 0.40 mmol) was added to a solution of compound **22**^4^ (100 mg, 0.13 mmol) in MeOH (2 mL) at room temperature. Solvents were evaporated and reaction was dissolved in EtOAc (10 mL), washed with saturated NaHCO_3_ solution (5 mL), dried over Na_2_SO_4_, and concentrated. After short column chromatography on silica-gel, boc-protected compound (100 mg, 0.12 mmol) was treated with thiocarbonyl diimidazole (0.042 g, 0.24 mmol) and DMAP (0.001 g, 0.012 mmol) in DCM (2.0 mL) at room temperature for 12 hours. Reaction mixture was evaporated and the crude product was purified through silica-gel column chromatography to afford pure product **23** (0.080 g, 71% over two steps) as a colorless oil. ^1^H NMR (500 MHz, CD_3_OD; mixture of rotamers) δ 8.36 (d, *J* = 18.3 Hz, 1H), 7.87 – 6.87 (m, 16H), 6.29 (dd, *J* = 8.8, 8.1 Hz, 1H), 5.39 – 4.92 (m, 4H), 4.81 (s, 1H), 4.79 – 4.46 (m, 5H), 4.40 – 3.74 (m, 4H), 3.21 – 3.18 (m, 1H), 3.12 (s, 3H), 3.04 (s, 3H), 1.74 – 1.57 (m, 2H), 1.36 (s, 9H), 1.15 (br s, 3H); ^13^C NMR (125 MHz, CD_3_OD) δ 183.6, 171.6, 162.9, 161.0, 157.4, 157.3, 156.8, 156.7, 156.5, 136.9, , 136.7, 136.6, 136.5, 134.9, 129.6, 129.5, 128.8, 128.7, 128.3, 128.2, 128.1, 127.9, 127.8, 127.7, 127.6, 127.5, 127.1, 118.4, 114.7, 114.5, 94.4, 92.5, 80.0, 77.2, 77.1, 76.6, 73.5, 72.6, 71.5, 67.3, 67.1, 67.0, 64.5, 64.4, 64.3, 64.2, 60.2, 57.2, 56.9, 56.8, 56.7, 56.6, 51.0, 39.0, 32.1, 31.8, 30.7, 30.6, 27.2, 20.0, 19.5, 13.1; HRMS (ESI) m/z calcd for C_47_H_57_FN_5_O_13_S [M+H]^+^, 950.3658; found, 950.3658.

1, 3*-N,N*-(bis-benzyloxycarbonyl)-6-deoxy*-*3’(*R*)-3’-[(4 fluoro)benzylaminomethyl)]dihydrospectinomycin (**24**)

Bu_3_Sn-H (0.113 mL, 0.42 mmol) and AIBN (0.04 mL, 0.008 mmol; 0.2M soln. in toluene) was added to a solution of imidazolyl thiocarbamate **23** (0.08 g, 0.08 mmol) in toluene (3.0 mL) at room temperature and heated at reflux for 30 minutes. The reaction mixture was cooled to room temperature, evaporated under high vacuum to give a crude product which was subjected to short column chromatography to give 6-deoxy compound. Trifluoroacetic acid (0.025 mL) was added to the solution of 6-deoxy compound (0.04 g) in DCM (1.0 mL) at room temperature. After 6 hours, the reaction mixture was diluted with DCM (10 mL), washed with saturated NaHCO_3_ solution (5 mL), dried over Na_2_SO_4_, and concentrated. Silica-gel column chromatography (Eluents: 0 - 3% CH_2_Cl_2_ in MeOH) yielded pure product **24** (0.025 g, 41% over 2 steps) as a colorless oil. ^1^H NMR (500 MHz, CD_3_OD; mixture of rotamers) δ 7.49 – 6.99 (m, 14H), 5.31 – 4.89 (m, 4H), 4.84 (br s, 1H), 4.56 (s, 1H), 4.50 (t, *J* = 10.7 Hz, 1H), 4.18 (s, 1H), 4.13 – 3.87 (m, 3H), 3.84 – 3.72 (m, 2H), 3.66 – 3.57 (m, 1H), 3.08 (s, 3H), 2.97 (s, 3H), 2.76 (d, *J* = 12.7 Hz, 1H), 2.23 – 2.08 (m, 1H), 1.75 – 1.53 (m, 3H), 1.17 (t, *J* = 4.1 Hz, 3H); ^13^C NMR (125 MHz, CD_3_OD) δ 163.2, 161.2, 157.3, 157.0, 136.8, 136.8, 134.6, 130.1, 130.0, 128.2, 127.6, 127.5, 127.4, 114.9, 114.7, 114.7, 95.9, 93.3, 93.2, 71.6, 71.6, 68.4, 66.9, 66.8, 66.7, 66.6, 60.1, 57.0, 41.9, 41.8, 30.5, 30.3, 27.1, 20.1, 13.1; HRMS (ESI) m/z calcd for C_38_H_47_FN_3_O_10_ [M+H]^+^, 724.3245; found, 724.3248.

**Table S1:** Minimum inhibitory concentration (mg/mL) of synthesized compounds against Gram (-) and Gram (+) strains.

| **Table S2**: Decomposition of the free energy contribution to the binding free energy onto the *M. tuberculosis* rRNA/RpsE complex determined using the MM/GBSA method in AMBER18. | | | | | |
| --- | --- | --- | --- | --- | --- |
| Nucleotide | Compound | E_VDW_  (kcal^-1^ mol^-1^) | E_electrostatic_  (kcal^-1^ mol^-1^) | E_dispersion_  (kcal^-1^ mol^-1^) | ΔG_total_  (kcal^-1^ mol^-1^) |
| G1064 | **1** | -0.05 $\pm$ 0.71 | -58.7 $\pm$ 1.35 | 53.1 $\pm$ 0.81 | -5.76 $\pm$ 0.73 |
|  | **5** | -1.03 $\pm$ 0.51 | -54.8 $\pm$ 1.03 | 52.0 $\pm$ 0.80 | -3.95 $\pm$ 0.34 |
|  | **9** | -0.21 $\pm$ 0.71 | -57.2 $\pm$ 1.30 | 52.1 $\pm$ 0.79 | -5.39 $\pm$ 0.73 |
|  | **2** | -0.44 $\pm$ 0.65 | -58.1 $\pm$ 1.37 | 52.6 $\pm$ 0.93 | -5.97 $\pm$ 0.76 |
|  | **6** | -2.08 $\pm$ 0.32 | -55.4 $\pm$ 1.22 | 53.9 $\pm$ 0.98 | -3.68 $\pm$ 0.44 |
|  | **10** | -0.36 $\pm$ 0.50 | -56.2 $\pm$ 1.24 | 51.1 $\pm$ 0.03 | -5.54 $\pm$ 0.66 |
| C1066 | **1** | -0.18 $\pm$ 0.59 | -55.7 $\pm$ 1.16 | 49.2 $\pm$ 0.64 | -6.78 $\pm$ 0.82 |
|  | **5** | -0.04 $\pm$ 0.61 | -54.8 $\pm$ 1.24 | 49.0 $\pm$ 0.61 | -5.96 $\pm$ 0.95 |
|  | **9** | -0.09 $\pm$ 0.56 | -51.1 $\pm$ 0.96 | 46.8 $\pm$ 0.61 | -4.45 $\pm$ 0.30 |
|  | **2** | -0.40 $\pm$ 0.54 | -53.5 $\pm$ 1.13 | 48.3 $\pm$ 0.63 | -5.71 $\pm$ 0.70 |
|  | **6** | -0.26 $\pm$ 0.49 | -56.7 $\pm$ 1.08 | 51.4 $\pm$ 0.68 | -5.69 $\pm$ 0.62 |
|  | **10** | -0.13 $\pm$ 0.50 | -49.7 $\pm$ 0.93 | 45.8 $\pm$ 0.60 | -4.13 $\pm$ 0.29 |
| G1068 | **1** | -1.35 $\pm$ 0.40 | -50.0 $\pm$ 1.03 | 45.2 $\pm$ 0.69 | -6.25 $\pm$ 0.57 |
|  | **5** | -1.46 $\pm$ 0.39 | -49.6 $\pm$ 1.03 | 44.4 $\pm$ 0.72 | -5.80 $\pm$ 0.53 |
|  | **9** | -1.23 $\pm$ 0.38 | -51.7 $\pm$ 0.97 | 46.2 $\pm$ 0.67 | -6.88 $\pm$ 0.48 |
|  | **2** | -1.56 $\pm$ 0.41 | -49.7 $\pm$ 1.06 | 45.9 $\pm$ 0.75 | -5.40 $\pm$ 0.50 |
|  | **6** | -0.64 $\pm$ 0.40 | -53.1 $\pm$ 1.09 | 47.1 $\pm$ 0.69 | -6.75 $\pm$ 0.63 |
|  | **10** | -1.41 $\pm$ 0.36 | -50.9 $\pm$ 1.03 | 46.0 $\pm$ 0.69 | -6.42 $\pm$ 0.48 |
| A1191 | **1** | -1.66 $\pm$ 0.31 | -28.7 $\pm$ 0.61 | 31.1 $\pm$ 0.58 | -1.83 $\pm$ 0.30 |
|  | **5** | -1.56 $\pm$ 0.29 | -27.5 $\pm$ 0.52 | 27.4 $\pm$ 0.48 | -1.72 $\pm$ 0.28 |
|  | **9** | -1.92 $\pm$ 0.14 | -27.9 $\pm$ 0.78 | 28.9 $\pm$ 0.62 | -1.02 $\pm$ 0.27 |
|  | **2** | -1.80 $\pm$ 0.27 | -30.2 $\pm$ 0.64 | 29.9 $\pm$ 0.54 | -2.26 $\pm$ 0.28 |
|  | **6** | -0.21 $\pm$ 0.44 | -27.3 $\pm$ 0.57 | 25.9 $\pm$ 0.43 | -1.71 $\pm$ 0.39 |
|  | **10** | -1.93 $\pm$ 0.11 | -29.5 $\pm$ 0.76 | 30.4 $\pm$ 0.59 | -1.10 $\pm$ 0.25 |
| C1192 | **1** | -1.03 $\pm$ 0.53 | -34.3 $\pm$ 0.86 | 31.1 $\pm$ 0.58 | -4.33 $\pm$ 0.46 |
|  | **5** | -1.07 $\pm$ 0.51 | -33.5 $\pm$ 0.84 | 23.6 $\pm$ 0.46 | -4.42 $\pm$ 0.46 |
|  | **9** | -1.11 $\pm$ 0.48 | -34.6 $\pm$ 0.86 | 31.3 $\pm$ 0.61 | -4.49 $\pm$ 0.45 |
|  | **2** | -1.30 $\pm$ 0.72 | -42.8 $\pm$ 1.12 | 37.3 $\pm$ 0.69 | -7.06 $\pm$ 0.68 |
|  | **6** | -2.66 $\pm$ 0.47 | -36.8 $\pm$ 1.08 | 36.7 $\pm$ 0.93 | -3.10 $\pm$0.47 |
|  | **10** | -1.23 $\pm$ 0.71 | -42.9 $\pm$ 1.07 | 37.4 $\pm$ 0.68 | -6.90 $\pm$ 0.64 |
| G1193 | **1** | -3.20 $\pm$ 0.22 | -24.5 $\pm$ 0.56 | 24.4 $\pm$ 0.47 | -3.56 $\pm$ 0.30 |
|  | **5** | -2.98 $\pm$ 0.23 | -23.6 $\pm$ 0.54 | 23.6 $\pm$ 0.46 | -3.26 $\pm$ 0.27 |
|  | **9** | -3.27 $\pm$ 0.23 | -24.6 $\pm$ 0.55 | 24.5 $\pm$ 0.47 | -3.57 $\pm$ 0.33 |
|  | **2** | -4.27 $\pm$ 0.50 | -27.9 $\pm$ 0.69 | 26.6 $\pm$ 0.55 | -5.99 $\pm$0.41 |
|  | **6** | -3.41 $\pm$ 0.50 | -28.5 $\pm$ 0.63 | 25.1 $\pm$ 0.48 | -7.12 $\pm$0.50 |
|  | **10** | -4.63 $\pm$ 0.53 | -28.8 $\pm$ 0.72 | 27.6 $\pm$ 0.56 | -6.24 $\pm$ 0.43 |
| Val56 | **1** | -0.17 $\pm$ 0.007 | 1.07 $\pm$ 0.09 | -0.94 $\pm$ 0.08 | -0.04 $\pm$ 0.03 |
|  | **5** | -0.14 $\pm$ 0.005 | 1.00 $\pm$ 0.09 | -0.89 $\pm$ 0.09 | -0.04 $\pm$ 0.02 |
|  | **9** | -0.18 $\pm$ 0.007 | 1.15 $\pm$ 0.09 | -1.01 $\pm$ 0.08 | -0.05 $\pm$ 0.03 |
|  | **2** | -1.21 $\pm$0.06 | 0.60 $\pm$ 0.07 | -0.93 $\pm$ 0.07 | -1.63 $\pm$ 0.09 |
|  | **6** | -0.80 $\pm$0.20 | 1.29 $\pm$ 0.09 | -1.43 $\pm$ 0.08 | -1.03 $\pm$ 0.20 |
|  | **10** | -1.34 $\pm$ 0.41 | 0.54 $\pm$ 0.08 | -0.95 $\pm$ 0.68 | -1.85 $\pm$ 0.89 |
| Values represent an average over the 20ns MD ensemble $\pm$ standard deviation. | | | | | |

**Table S3**: Whole cell accumulation (mM) assay in *E. coli*.(wild type) and Δtolc strains.

| Compiled Accumulation data (uM) | | |
| --- | --- | --- |
| Compounds | BW25113 | △tolC |
| **1**-parent | 0.5 +0.1 | 0.5 +0.1 |
| **1**-Hydrate | 0.5 +0.1 | 0.6 +0.2 |
| **2** | 5.5 +0.8 | 83 +4 |
| **3** | 40 +1 | 59 +2 |
| **4** | 6.0 +0.7 | 8 +3 |
| **5**-parent | 1.1 +0.3 | 1.0 +0.3 |
| **5**-Hydrate | 1.2 +0.1 | 0.85 +0.09 |
| **6** | 10.0 +0.6 | 15.7 +0.6 |
| **7** | 51 +6 | 62 +2 |
| **8** | 1.29 +0.06 | 1.8 +0.4 |
| **9**-Parent | Undetectable | Undetectable |
| **9**-Hydrate | 0.4 +0.1 | 0.19 +0.04 |
| **10** | 5.6 +0.5 | Not determined |
| **11** | 1.76 +0.06 | 0.37 +0.05 |


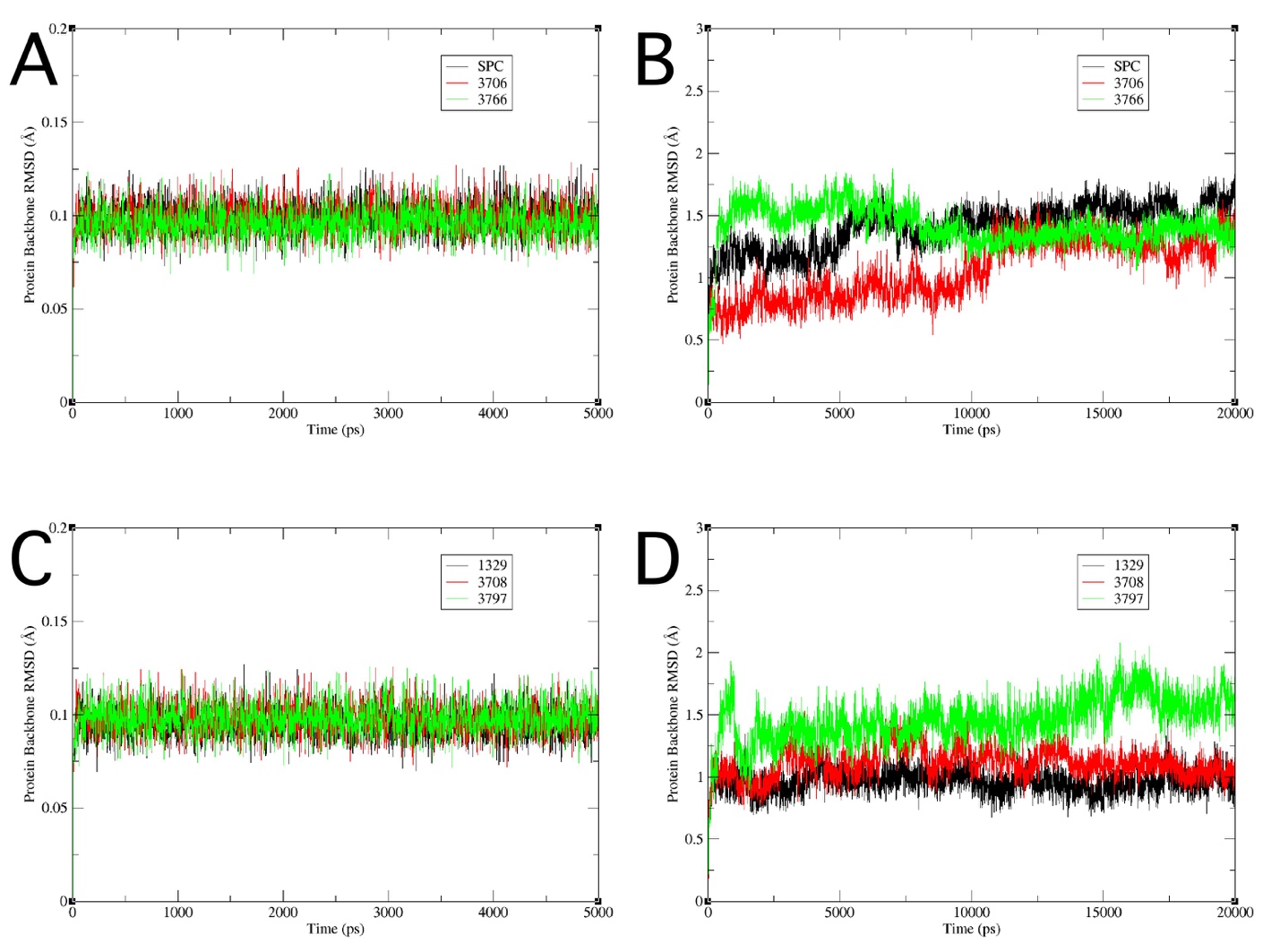


**Figure S1.** The protein backbone RMSD during the (A,C) 500 ps equilibration step and (B,D) 20 ns MD simulation of analogs.


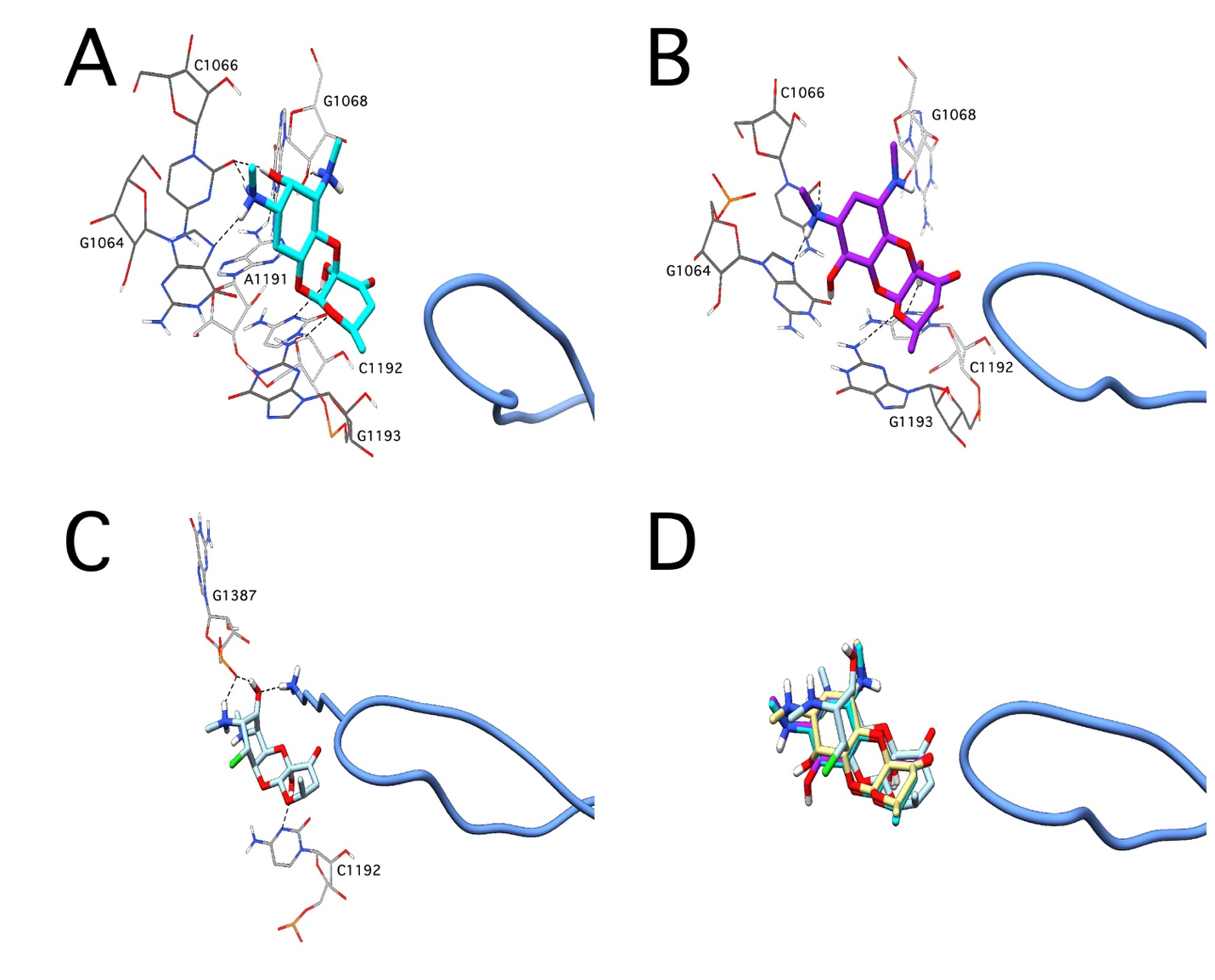
**Figure S2.** The molecular docking of deoxyspectinomycin analogs onto the *M. tuberculosis* rRNA/RpsE complex by Glide in Schrödinger. The docked conformation with the highest Glide score for A) **5** (-7.32 kcal mol^-1^)**,** B) **9** (-6.57 kcal mol^-1^)**,** and C) **8** (-6.54 kcal mol^-1^) is shown. The protein is shown as a blue ribbon with nucleotides interacting with the analog are labeled. The hydrogen bonding interactions are depicted as dashed lines. D) An overlay of **1** (-8.87 kcal mol^-1^, yellow), **5** (cyan), **9** (purple), and **8** (light blue) from molecular docking.


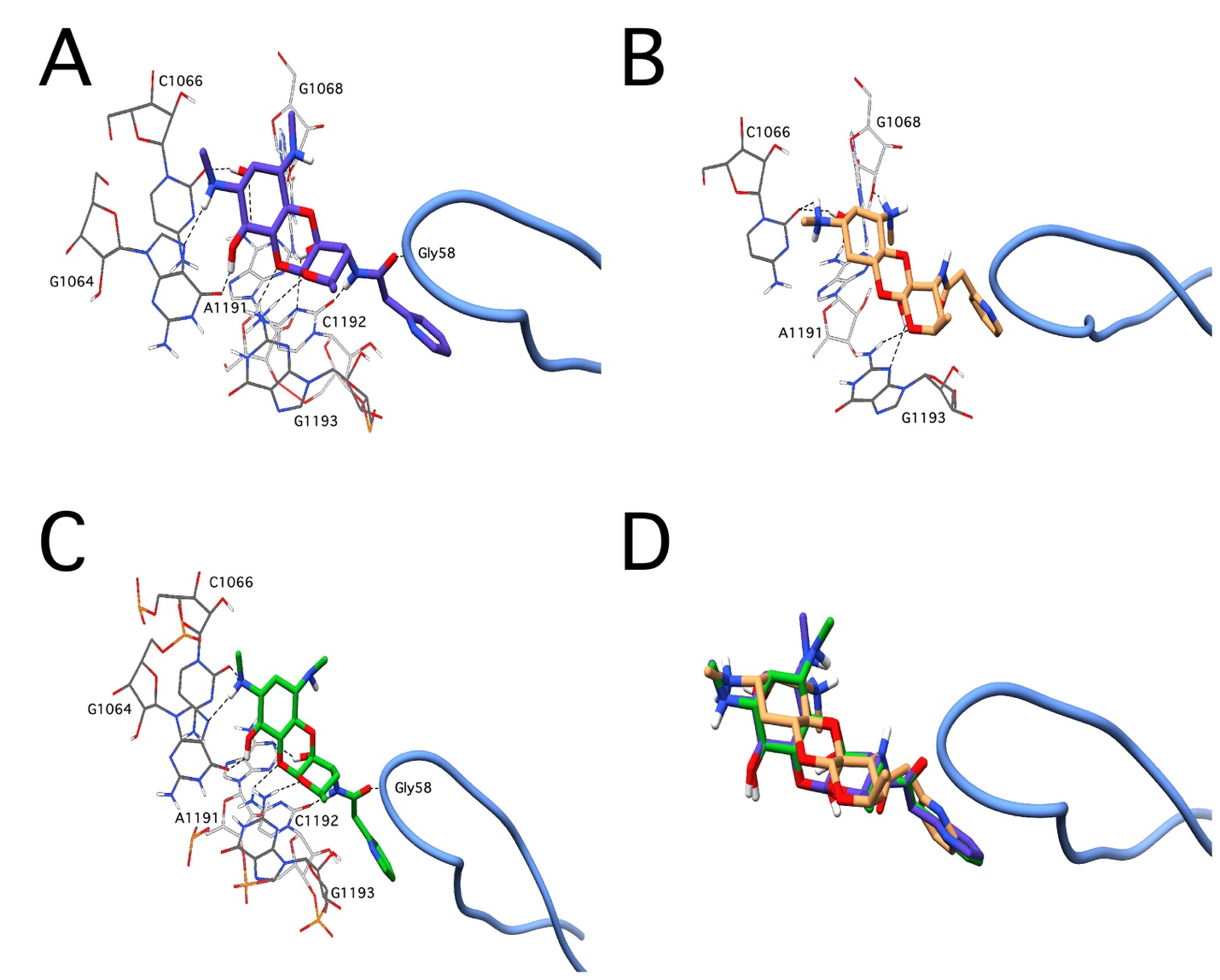
**Figure S3.** The molecular docking of spectinamide and deoxy analogs onto the *M. tuberculosis* rRNA/RpsE complex by Glide in Schrödinger. The docked conformation with the highest Glide score for A) **2** (-10.43 kcal mol^-1^), B) **6** (-8.25 kcal mol^-1^), and C) **10** (-9.34 kcal mol^-1^) is shown. The protein is shown as a blue ribbon with nucleotides interacting with the analog are labeled. The hydrogen bonding interactions are depicted as dashed lines. D) An overlay of **2** (purple), **6** (orange), and **10** (green) from molecular docking.


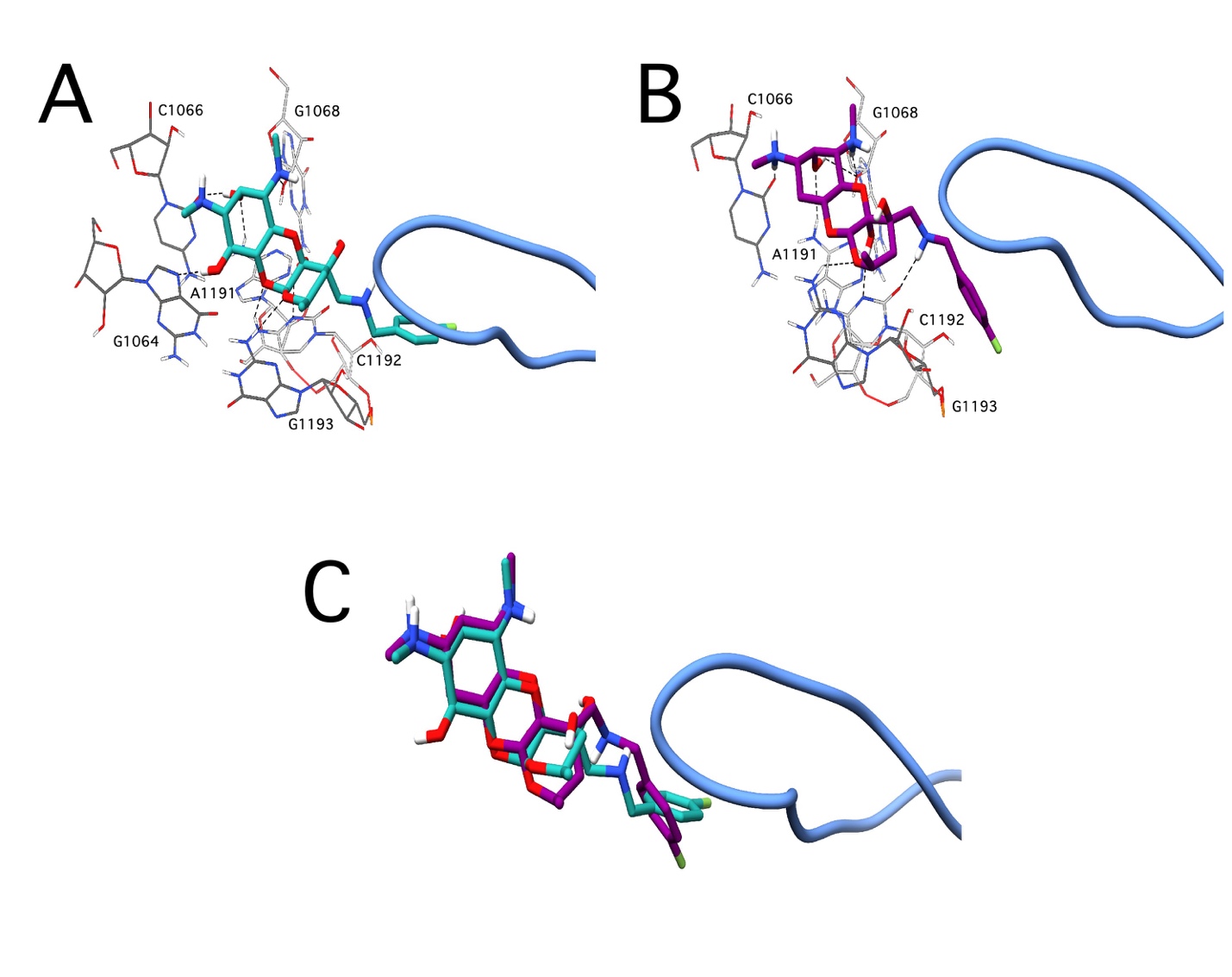
**Figure S4.** The molecular docking of amSPC and the deoxy analog onto the *M. tuberculosis* rRNA/RpsE complex by Glide in Schrödinger. The docked conformation with the highest Glide score for A) **3** (-9.17 kcal mol^-1^) and B) **7** (-7.29 kcal mol^-1^) is shown. The protein is shown as a blue ribbon with nucleotides interacting with the analog are labeled. The hydrogen bonding interactions are depicted as dashed lines. C) An overlay of **3** (teal) and **7** (dark magenta) from molecular docking.


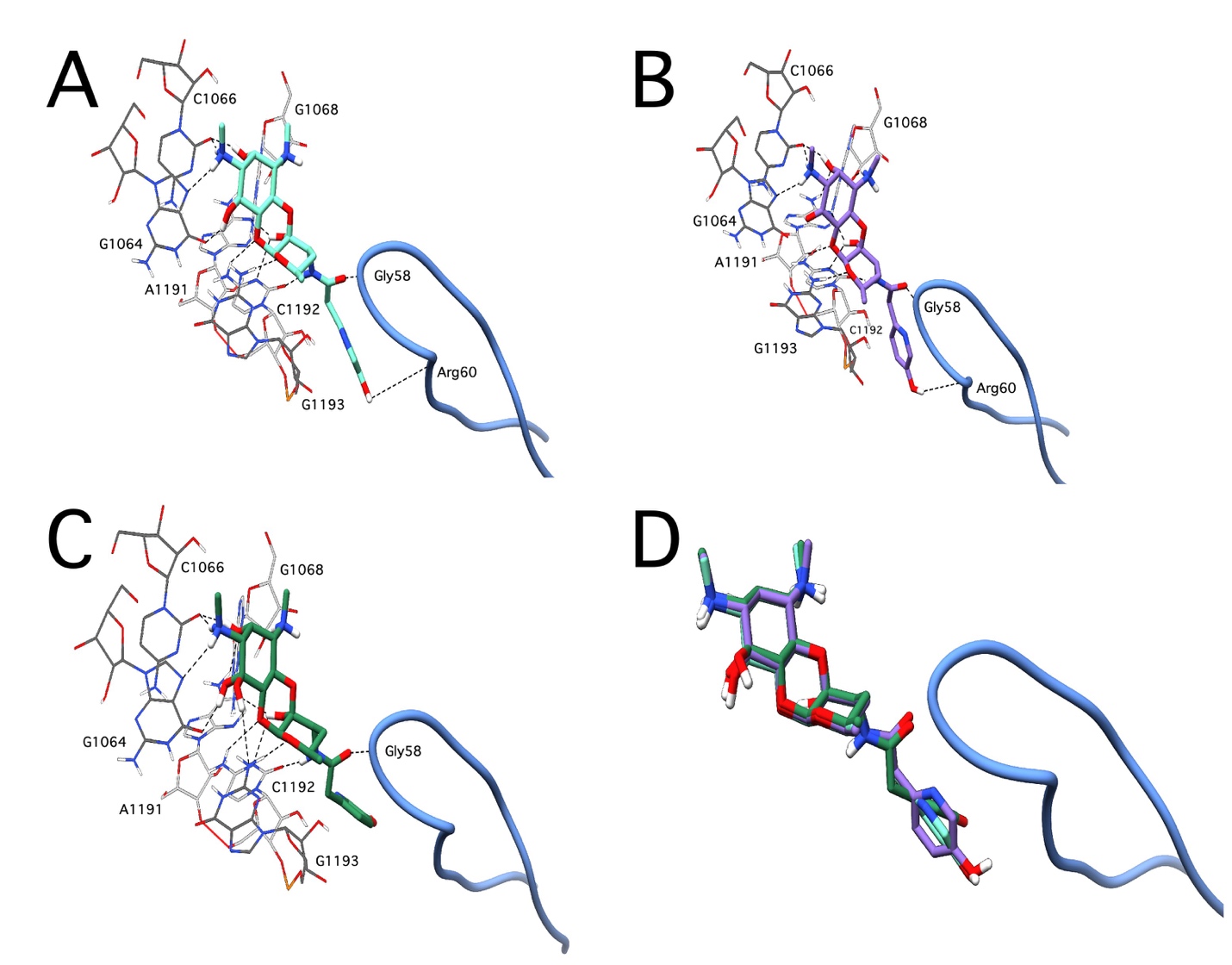
**Figure S5.** The molecular docking of spectinamide and the 6-dehydrospectinamide onto the *M. tuberculosis* rRNA/RpsE complex by Glide in Schrödinger. The docked conformation with the highest Glide score for A) **4** (-10.54 kcal mol^-1^), B) **11** (-10.82 kcal mol^-1^), and C) **12** (-9.20 kcal mol^-1^) is shown. The protein is shown as a blue ribbon with nucleotides interacting with the analog are labeled. The hydrogen bonding interactions are depicted as dashed lines. D) An overlay of **4** ( cyan), **11** ( purple), and **12 (** dark green) from molecular docking.

References:

1. Foley L, Weigele M. Spectinomycin chemistry. 1. Characterization of a 5a,9a-epi-4(R)-dihydrospectinomycin derivative. *The Journal of Organic Chemistry.* 43(22):4355-4359 (1978)

2. Maier R, Woitun E, Reuter A, Reuter W, Wetzel B. Modification of spectinomycin. 1. Synthesis of 4-aminospectinomycins. *The Journal of antibiotics.* 34(1):16-21 (1981)

3. Foley L, Lin JT, Weigele M. Preparation of 7-deoxyspectinomycin and 7-deoxy-8-epi-4(R)-dihydrospectinomycin. *The Journal of antibiotics.* 32(4):418-419 (1979)

4. Bruhn DF, et al. Aminomethyl spectinomycins as therapeutics for drug-resistant respiratory tract and sexually transmitted bacterial infections. *Science translational medicine.* 7(288):288ra275 (2015)

^1^H NMR (500 MHz, D_2_O) Spectrum of 6-Deoxyspectinomycin dihydrochloride (**5**)


^1^3C NMR (125 MHz, D_2_O) Spectrum of 6-Deoxyspectinomycin dihydrochloride (**5**)
^1^H NMR (500 MHz, D_2_O) Spectrum of 6-Deoxy-3'-deoxy-3'-dihydro-3'(*R*)-[(pyridin-2-yl)acetylamino]spectinomycin dihydrochloride (**6**)

^13^C NMR (125 MHz, D_2_O) Spectrum of 6-Deoxy-3'-deoxy-3'-dihydro-3'(*R*)-[(pyridin-2-yl)acetylamino]spectinomycin dihydrochloride (**6**)

^1^H NMR (500 MHz, D_2_O) Spectrum of 6-Deoxy-3’-dihydro-3'(*R*)-[(4-fluorobenzyl)aminomethyl]spectinomycin trihydrochloride (**7**)

^13^C NMR (125 MHz, D_2_O) Spectrum of 6-Deoxy-3’-dihydro-3'(*R*)-[(4-fluorobenzyl)aminomethyl]spectinomycin trihydrochloride (**7**)

^1^H NMR (500 MHz, D_2_O) Spectrum of 2-Deoxyspectinomycin dihydrochloride (**9**)

^13^C NMR (125 MHz, D_2_O) Spectrum of 2-Deoxyspectinomycin dihydrochloride (**9**)

^1^H NMR (500 MHz, D_2_O) Spectrum of 2-Deoxy-3'-deoxy-3'-dihydro-3'(*R*)-[(pyridin-2-yl)acetylamino]spectinomycin dihydrochloride (**10**)

^13^C NMR (125 MHz, D_2_O) Spectrum of 2-Deoxy-3'-deoxy-3'-dihydro-3'(*R*)-[(pyridin-2-yl)acetylamino]spectinomycin dihydrochloride (**10**)
